# Species identification and molecular typing of human *Brucella* isolates from Kuwait

**DOI:** 10.1371/journal.pone.0182111

**Published:** 2017-08-11

**Authors:** Abu S. Mustafa, Nazima Habibi, Amr Osman, Faraz Shaheed, Mohd W. Khan

**Affiliations:** 1 OMICS Research Unit and Research Core Facility, Heath Sciences Centre, Kuwait University, Jabriya, Kuwait; 2 Department of Microbiology, Faculty of Medicine, Heath Sciences Centre, Kuwait University, Jabriya, Kuwait; Universidad Nacional de la Plata, ARGENTINA

## Abstract

Brucellosis is a zoonotic disease of major concern in Kuwait and the Middle East. Human brucellosis can be caused by several *Brucella* species with varying degree of pathogenesis, and relapses are common after apparently successful therapy. The classical biochemical methods for identification of *Brucella* are time-consuming, cumbersome, and provide information limited to the species level only. In contrast, molecular methods are rapid and provide differentiation at intra-species level. In this study, four molecular methods [16S rRNA gene sequencing, real-time PCR, enterobacterial repetitive intergenic consensus (ERIC)-PCR and multilocus variable-number tandem-repeat analysis (MLVA)-8, MLVA-11 and MLVA-16 were evaluated for the identification and typing of 75 strains of *Brucella* isolated in Kuwait. 16S rRNA gene sequencing of all isolates showed 90–99% sequence identity with *B*. *melitensis* and real-time PCR with genus- and species- specific primers identified all isolates as *B*. *melitensis*. The results of ERIC-PCR suggested the existence of 75 ERIC genotypes of *B*. *melitensis* with a discriminatory index of 0.997. Cluster classification of these genotypes divided them into two clusters, A and B, diverging at ~25%. The maximum number of genotypes (n = 51) were found in cluster B5. MLVA-8 analysis identified all isolates as *B*. *melitensis*, and MLVA-8, MLVA-11 and MLVA-16 typing divided the isolates into 10, 32 and 71 MLVA types, respectively. Furthermore, the combined minimum spanning tree analysis demonstrated that, compared to MLVA types discovered all over the world, the Kuwaiti isolates were a distinct group of MLVA-11 and MLVA-16 types in the East Mediterranean Region.

## Introduction

Human brucellosis, a common zoonotic disease, is a neglected, under-recognized infection of widespread geographic distribution and globally about 500,000 cases occur annually [1]. The highest incidence of human brucellosis is recorded in the Middle East and Central Asia [2, 3]. It is among the most commonly reported infectious diseases in Kuwait and the other countries of Gulf Cooperation Council (GCC) [4, 5]. The organisms causing brucellosis belong to genus *Brucella*, and the disease in humans is primarily caused by three species, i.e. *B*. *melitensis*, *B*. *suis* or *B*. *abortus* [1].

The natural reservoir of *Brucella* and the source of infection in humans are infected domestic animals, including cattle, sheep, goats, camels as well as wild animals [6, 7]. The transmission of *Brucella* from animals to humans normally occurs via direct contact with the infected animals, and consumption of unpasteurized milk and milk products [8]. Human brucellosis is a debilitating and disabling illness, and has major economic ramifications due to time lost by patients from normal daily activities [8]; and livestock infections have substantial socioeconomic impact [9, 10]. *Brucella* spp. is highly infectious through aerosol route as well, making it an attractive pathogen to be used as a potential agent for biological warfare [11, 12].

Although, *Brucella* species differ widely in host preference, phenotypic characteristics, and pathogenicity; they are genetically homogeneous, with more than 99% nucleotide sequence identity, as demonstrated by comparative whole genome analysis [13]. Therefore, the classical molecular methods, e.g. DNA hybridization, have failed to differentiate among various species of *Brucella* [13]. The identification of *Brucella* spp. became possible by the development of high resolution molecular methods, e.g. singleplex and multiplex PCRs, etc. [14–16]. Currently, a rapid identification of *Brucella* is possible by performing the 16S rRNA gene sequencing [17, 18], and real-time PCR-based high resolution melt (HRM) analysis for genus and species identification [19, 20].

The relapse of clinical symptoms after 2–40 years of apparently successful treatment has been reported in 5–40% of brucellosis patients [21–25]. However, it is not possible to differentiate between relapse and reinfection because the classical techniques of culture, serology and PCRs with genus and species-specific primers can only identify *Brucella* at species and biovar levels [26]. To differentiate between relapse and reinfection, further genetic identification of *Brucella* at genotype level using relevant molecular typing methods is essential [27–29]. In addition, molecular methods for subtyping isolates are necessary for allowing epidemiological surveillance, contact tracing, introduction of new strains, gauging the success of eradication programs and investigating outbreaks in countries endemic for brucellosis [30–34]. The technique of enterobacterial repetitive intergenic consensus sequence (ERIC)-PCR has been used widely to determine the bacterial genotypes at the subspecies level [35, 36], including *Brucella* species [37, 38]. The ERIC-PCR is a relatively simple technique, as compared to the other PCR-based genotyping assays, because a pair of random primers anneal at non-specific places at the whole genome level to produce strain-specific band patterns [35, 36]. This method has the ability to differentiate between individual *Brucella* isolates [37, 38].

In 2006, a highly discriminatory method for genotyping *Brucella*, based on multilocus variable-number tandem-repeat analysis (MLVA), was developed by Le Fleche and co-workers [39]. This method has been used to type various species and strains of *Brucella* with fine-scale resolution of closely related isolates [27–34, 40, 41]. The MLVA-16 system comprises of eight minisatellite or Panel 1 markers (Bruce06, Bruce08, Bruce11, Bruce12, Bruce42, Bruce43, Bruce45, and Bruce55) and eight complementary microsatellite or Panel 2 markers divided into panel 2A (Bruce18, Bruce19, and Bruce21) and panel 2B (Bruce04, Bruce07, Bruce09, Bruce16, and Bruce30). The Panel 1 markers (MLVA8) are considered suitable for *Brucella* species identification and the Panel 2B markers for subspecies differentiation [27, 40]. By using MLVA analysis, it has been shown that *Brucella* isolates from different patients in an outbreak or from the same patient before the start of therapy and after relapse exhibited identical genotypes [27, 29]. Because of its rapidity, highly discriminatory power and reproducibility, it has been suggested that MLVA assay can be useful in epidemiological trace-back analysis of *Brucella* infections with the potential to advance surveillance and control of human brucellosis [27].

In this study, we have identified the predominant *Brucella* spp. infecting humans in Kuwait by 16S rRNA gene sequencing and real-time PCR HRM analysis, and genotyped the isolates using ERIC-PCR and MLVA techniques. The genetic relatedness of Kuwaiti strains with the strains isolated internationally was determined by comparing the MLVA types using minimum spanning tree (MST) analysis.

## Materials and methods

### *Brucella* strains and DNA isolation

A total of 75 *Brucella* strains (BRU001-BRU118) isolated from 75 patients and cultured on plates at the Clinical Microbiology Laboratories of Infectious Diseases, Mubarak Al-Kabeer, Farwaniya and Amiri Hospitals in Kuwait. A loopful of bacterial colonies from each plate was suspended into 1 ml phosphate buffered saline (pH 7.0) and heated at 95°C for 10 min in a water bath. The genomic DNA was isolated from the heated specimens using the QIAamp DNA Mini Kit (Qiagen, Valencia, CA) according to the manufacturer’s instructions. The quantities and purities of isolated DNA were determined using an Epoch Spectrophotometer (Biotek, Winooski, VT) and Qubit Fluorometer (Qubit dsDNA BR Assay Kit, Life Technologies, Carlsbad, CA). The isolated DNA was stored at −80°C until further use.

### Amplification and sequencing the 16S rRNA gene

A 500 bp region of 16S rRNA gene from *Brucella* genomic DNA was amplified using the MicroSeq^®^ 500 16S rDNA PCR kit (Applied Biosystems, Grand Island, NY) according to the manufacturer’s instructions. In brief, the PCR reaction mixtures contained 15 μl of genomic DNA (25 ng) and 15 μl of 2x master mix (the universal primers, AmpliTaq^®^ gold DNA Polymerase, Buffer, MgCl2 and dNTP mix) from the kit, and the PCR was performed in a GeneAmp^®^ PCR System 9700 (Applied Biosystems, Grand Island, NY). PCR mixtures containing positive (DNA isolated from *Escherichia coli*) and negative controls (nuclease-free water), included in the kit, were also prepared. The conditions used for target amplification were as follows: 95°C for 10 min, 30 cycles of 95°C for 30 s, 60°C for 30 s, and 72°C for 45 s, and a final extension step at 72°C for 10 min. The PCR products were visualized on a DNA 1000 gel in a Bioanalyzer (Agilent 2100, Santa Clara, CA). The amplified products were purified by adding 2μl of ExoSAP-IT^®^ to 5μl of the PCR product and incubated for 15 min at 37°C, followed by heat inactivation of the enzyme at 80°C for 15 min. Cycle sequencing was performed with the purified PCR products using the MicroSeq^®^ 500 sequencing kit (BigDye^®^ Terminator v1.1 chemistry) as per the kit protocol. The thermal cycler was programmed at 96°C for 10 s, 50°C for 5 s and 60°C for 4 min for 25 cycles. The extension products were purified with Performa^®^ DTR Gel Filtration Cartridges (Edge Biosystems, Gaithersburg, MD) and sequenced on an ABI 3130xl automated Genetic Analyzer (Applied Biosystems, Foster City, CA). The ABI files were opened with the Sequencing Analysis software (Applied Biosystems, Foster City, CA) for the quality assessment. DNA sequences in fasta format were further uploaded in the BioNumerics version 7.5 software (Applied Maths, Sint-Martens-Latem, Belgium) and submitted to the Ribosomal Database Project (RDP) [42] via the RDP plugin for similarity scores and percent identity calculation. The DNA sequence data have been submitted to the GenBank database under accession numbers MF164063 to MF164137.

### Real-time PCR and HRM analysis

The real-time PCR assays were performed using *Brucella* genus-specific forward (O1: 5’-TCCGCAAGCTTCAAGCCTTCTATC-3’) and reverse (O2: 5’-GGCGTGTCTGCATTCAACGTAACC-3’) primers [43], and *B*. *melitensis*-specific forward (BF: 5’-CATGCGCTATGTCTGGTTAC-3’) and reverse (BMR: 5’- AGTGTTTCGGCTCAGAATAAT-3’) primers [44]. The real-time PCR mixture was prepared by adding 5 μl of 10× PCR Buffer II, 0.7 μM final concentration of the forward and reverse primers (1μl each), 10 ng of DNA template (2 μl), SYBR Green I (2 μl), MgCl2 (2.4 μl) and nuclease free water (Qiagen, Germany) to a total volume of 25 μl per reaction. The real-time PCR was performed in a Light Cycler^®^ 2.0 (Roche Diagnostics GmbH, Mannheim, Germany) with an initial denaturation step of 95°C for 10 min, followed by 35 cycles of 95°C for 15 s and 65°C for 10 s, with acquisition of data at 72°C for 15 s in the green channel (excitation at 470 nm and detection at 510 nm). After amplification, the HRM analysis was performed between 65°C and 95°C at the rate of 0.1°C.

### ERIC-PCR

ERIC-PCR was performed with the primers ERIC1R (5′-ATGTAAGCTCCTGGGGATTCAC-3’) and ERIC2 (5′-AAGTAAGTGACTGGGGTGAGCG-3’) as described previously [36]. In brief, the PCR reaction mixtures contained 6 μl of 5x HOT FIREPol^®^ Blend Master mix (Solis BioDyne, Estonia), 10 ng DNA, 0.15 μM of each primer in a total volume of 30 μl. PCR conditions were 95°C for 12 min; 45 cycles of 95°C for 45 sec, 35°C for 1 min and 70°C for 10 min; and a final step at 70°C for 20 min. The amplification products were visualized on a DNA 7500 gel by the Bioanlayzer (Agilent 2100) and the band patterns were analyzed by the BioNumerics version 7.5 software (Applied Maths). In-house validation of the assay was done for the reproducibility employing three technical and biological replicates. Clustering was performed using the Dice similarity coefficient (optimization of 0.5%, tolerance of 1% and active zones of 10–78%) and unweighted- pair group method using arithmetic averages (UPGMA). Clusters were further classified based on *ca*. 80% of similarity [36]. The Discriminatory Index (DI) was calculated by the online tool (insilico.ehu.es/mini_tools/discriminatory_power/index.php).

### MLVA typing

Amplification of the 16 VNTR loci for MLVA typing was performed, using primer pairs specific for each locus, according to the methods described previously [27–34]. In brief, PCR was performed in a total volume of 20 μl containing 1 ng of DNA, 1× PCR Master Mix (Solis BioDyne, Tartu, Estonia), and 0.6 μM of each forward and reverse primers. Thermal cycling was performed in a GeneAmp 9700 Thermal Cycler (Applied Biosystems) by initial heating at 95°C for 3 min, followed by 30 cycles of denaturation at 95°C for 30 s, annealing at 60°C for 30 s and extension at 72°C for 50 s. The PCR products were visualized on a DNA 1000 gel by the Bioanlayzer (Agilent 2100) for accurate band size estimation. DNA 1000 ladder (100–1000 bp fragment size) from the Agilent kit was used as a control to estimate the band sizes of PCR products. Band sizes were converted into number of tandem repeat units according to the 2013 *Brucella* allele assignment table (version 3.6, available at http://mlva.u-psud.fr). The data set was submitted to the Brucella MLVA Database (http://mlva.u-psud.fr) for genotype identification on the basis of Panel 1 (MLVA8-type), Panel 1+2A (MLVA11-type) and Panel 1+2A+2B (MLVA16-type). All isolates were identified at *Brucella* spp. level by the MLVA8 typing scheme. The repeat numbers were imported to BioNumerics 7.5 software as character sets for cluster analysis based on categorical coefficients and UPGMA on similar weight basis. The discriminatory power of MLVA markers were calculated by the Hunter and Gaston Diversity Index (HGDI) via the online tool V-DICE available at the HPA website (http://www.hpabioinformatics.org.uk/cgi-bin/DICI/DICI.pl).

In order to determine the genetic relatedness of *B*. *melitensis* strains of our study with the strains available in the MLVA database, the VNTR copy numbers of 827 MLVA types from four major clades of the world (Africa, Americas, and East and West Mediterranean Regions) were downloaded from the MLVA database. Neighbor-joining minimum spanning trees were constructed from the datasets of the downloaded strains and the strains from Kuwait using BioNumerics 7.5 software.

## Results

### 16S rRNA gene sequencing and real-time PCR

PCR of the 16S rRNA genes of all the 75 isolates showed bands at about 500 bp. The positive control containing *E*. *coli* DNA also produced a band at ~500 bp, whereas the negative control did not produce any bands (S1 Fig). The nucleotide lengths of the PCR products obtained after 25 cycles of sequencing PCR were ~500 bp long (S1 Table). The quality value of each base call was >20 and an overall specimen score ranging from 26–46 was obtained for all the samples through the Sequencing Analysis software. Submission of all the 75 sequences to RDP database via BioNumerics identified the isolates as *B*. *melitensis*, exhibiting similarity values of 0.827 to 1.000 and 90–99% sequence identity (S1 Table). The results of real-time PCR assays with genus- and specific-specific primers confirmed that that all isolates belonged to genus *Brucella*, and species *B*. *melitensis* (data not shown).

### ERIC-PCR

**Fig 1:**
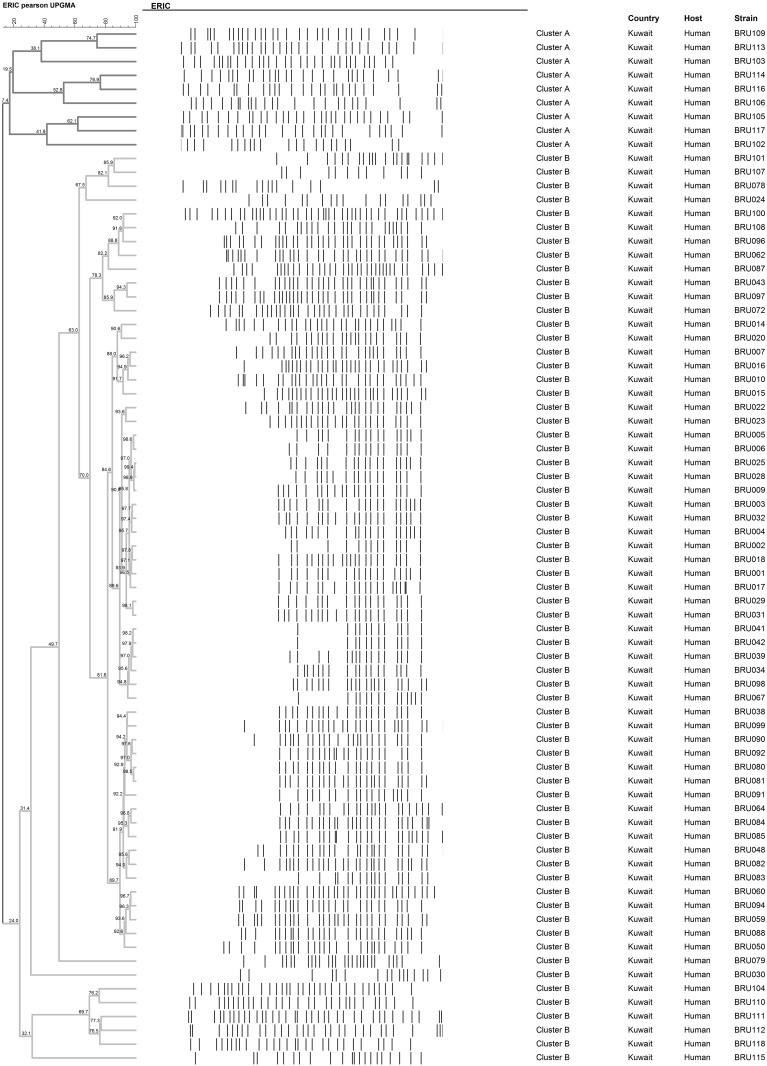
Cluster analysis of ERIC fingerprints of 75 strains of *B*. *melitensis* isolated in Kuwait. Band profiles of each strain are shown corresponding with the lines of the dendrogram. Two major clusters A and B (diverging ~ 25%) are demarcated by shades of grey. The ERIC genotype, strain Id, country and host are represented in the columns.

The ERIC-PCR products were well resolved in the Agilent Bioanlayzer, yielding peaks at corresponding bands (S2 Fig). The ERIC primers generated polymorphic band patterns in all the 75 isolates with varying numbers (13 to 39) and sizes of bands (73 bp to 5000 bp). The in-house validation results indicated the reproducibility of the assay by producing bands of similar intensities and length in all the technical and biological replicates (S3 Fig). The band profiles produced a dendrogram of 75 branches (Fig 1), which suggested that each sample was of a unique type. The DI calculated based on this finding was 0.997 confirming the high discriminatory power of the technique. The cluster classification based on ~ 80% similarity divided all the ERIC genotypes into two major clusters, A and B. Cluster A consisted of 9 ERIC genotypes (A1-A9) corresponding to 9 individual strains. Cluster B comprised of 13 sub clusters (B1-B13) amongst which B5 formed the largest cluster with 51 strains (68% of total population) sharing more than 80% similar ERIC profiles (Fig 1), followed by B3 consisting of 5 strains. Clusters B1 and B4 were composed of 3 strains and the remaining B2, B6, B7, B8 and B9 had only one strain each.

### MLVA typing

PCR amplification products were obtained with the sixteen MLVA primers in the presence of DNA from all 75 *Brucella* strains. However, varying number of alleles were detected, which ranged from 11 alleles for Bruce04 to only one allele for Bruce45 (Table 1). Overall, MLVA primers exhibited high discriminatory power, as observed from the HGDI value of 0.942. However, the individual set of primers varied in HGDI values ranging from as low as 0.000 (Bruce45) to as high as 0.888 (Bruce04) (Table 1).

**Table 1:** Allele frequencies and diversity indices of MLVA-16 primers for 75 *Brucella* strains isolated in Kuwait.

Locus	K^a^	VNTRs	HGDI^b^	Confidence Interval^c^	Max (pi)^d^
**MLVA-16/ Panel 1+ 2A+ 2B**	23	1,2,3,4,5,6,7,8,9,10,11,12,13,14,16,20,38,41,43,44,45,46,49	0.942	0.927–0.956	0.120
**MLVA-11/Panel 1+2A**	20	1,2,3,4,5,6,7,8,9,10,11, 12,13,38,41,43,44,45,46,49	0.881	0.859–0.903	0.173
**MLVA-8/ Panel 1**	9	1,2,3,4,5,6,11,12,13	0.605	0.534–0.676	0.520
Bruce06	4	1,2,3,4	0.131	0.027–0.235	0.932
Bruce08	2	5,6	0.027	0.000–0.078	0.986
Bruce11	3	2,3,4	0.080	0.000–0.164	0.959
Bruce12	3	11,12,13	0.105	0.010–0.200	0.946
Bruce42	4	1,2,3,4	0.488	0.404–0.573	0.649
Bruce43	3	1,2,3	0.224	0.102–0.346	0.878
Bruce45	1	3	0.000	0.000–0.093	1.000
Bruce55	3	2,3,5	0.154	0.045–0.263	0.919
**Panel 2A**	13	5,6,7,8,9,10,38,41,43,44,45,46,49	0.859	0.826–0.893	0.253
Bruce18	6	5,6,7,8,9,10	0.595	0.539–0.652	0.459
Bruce19	7	38,41,43,44,45,46,49	0.796	0.758–0.833	0.284
Bruce21	4	6,7,8,9	0.302	0.181–0.423	0.824
**Panel 2B**	13	4,5,6,7,8,9,10,11,12,13,14,16,20	0.939	0.927–0.950	0.120
Bruce04	11	5,6,7,8,9,10,11,12,13,14,16	0.888	0.865–0.912	0.189
Bruce16	9	4,5,6,7,8,9,10,11,12	0.840	0.808–0.871	0.257
Bruce09	7	4,5,6,7,8,12,20	0.709	0.649–0.768	0.405
Bruce07	5	4,5,6,7,9	0.578	0.483–0.672	0.595
Bruce30	5	5,6,7,8,10	0.352	0.221–0.482	0.797

^**a**^**K** = Number of different repeats present.

^**b**^**HGDI** (for VNTR data) = A measure of the variation of the number of repeats at each locus. Ranges from 0.0 (no diversity) to 1.0 (complete diversity).

^**c**^**Confidence Interval** = Precision of the Diversity Index, expressed as 95% upper & lower boundaries.

^**d**^**Max(pi)** = Fraction of samples that have the most frequent repeat number in this locus (range 0.0 to 1.0).

**Fig 2:**
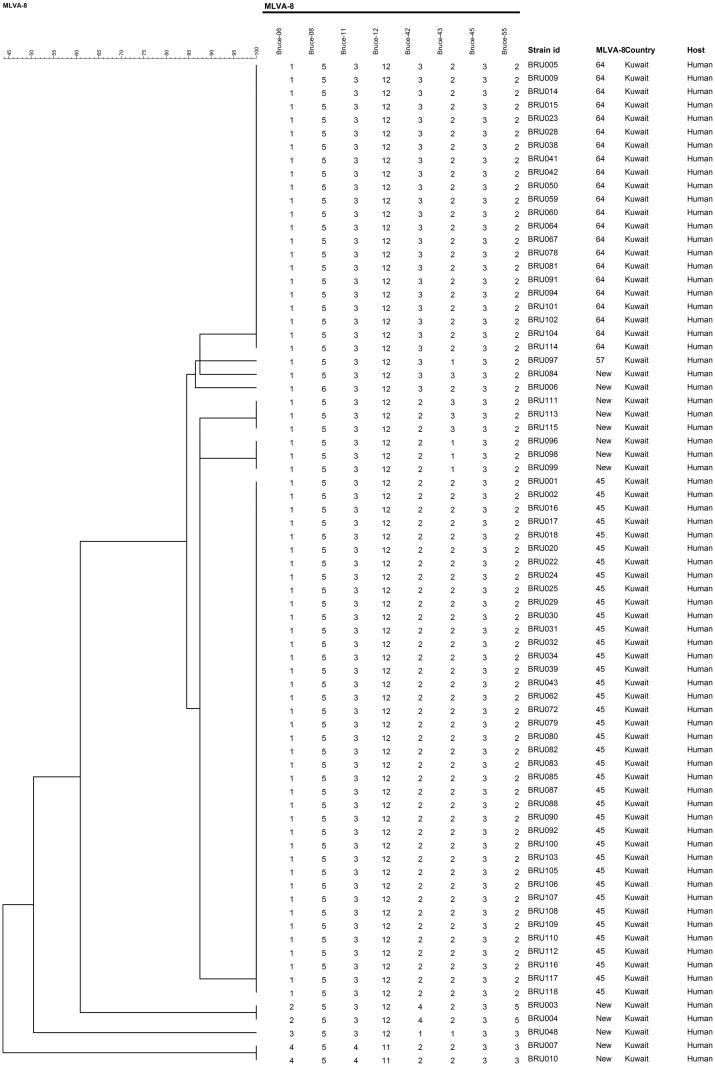
Dendrogram representing the major and novel *B*. *melitensis* strains (MLVA-8) identified in Kuwait. MLVA-8 type was assigned on the basis of VNTR copy numbers generated and queried on the MLVA database. The MLVA-8 genotype is presented in the columns along with the strain id, country and host. The novel genotypes are prefixed with the letter K.

The VNTR numbers derived through the banding pattern obtained from Panel 1 (MLVA-8) markers matched with *B*. *melitensis* in the MLVA database. Furthermore, the Panel 1 markers divided the 75 isolates into ten MLVA-8 genotypes, three of which have been reported previously and seven genotypes that were unique (Fig 2). Among the previously reported MLVA-8 genotypes, the genotype number 45 was the predominant type in Kuwait (40 strains), followed by 64 (22 strains) and 57 (one strain). Seven unique genotypes, not reported in the MLVA-8 database, were named as K1, K2, K4, K5, K6, K7 and K8 (Fig 2). Two strains each belonged to K1 and K5 genotypes, and three strains each in K2 and K8; whereas the remaining three unique genotypes had only one strain, *i*.*e*. K7-BRU084, K2-BRU115, K4-BRU006 (Fig 2).

**Fig 3:**
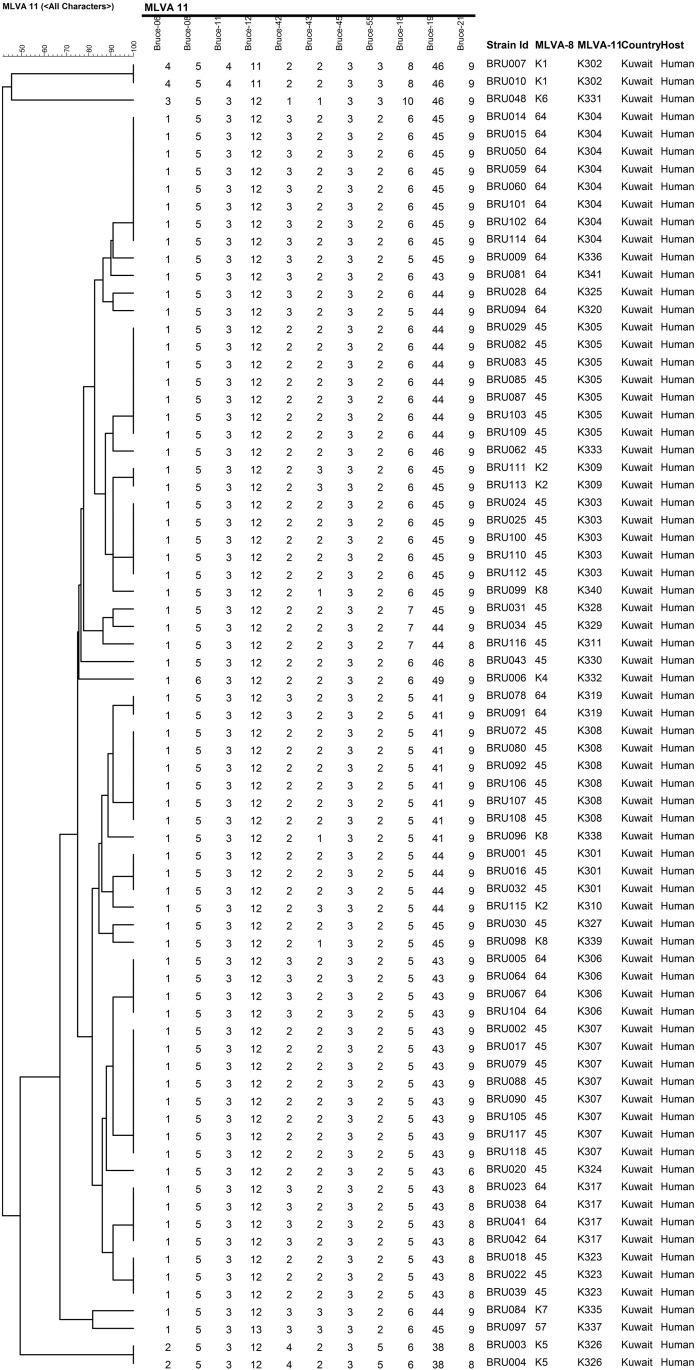
Cluster analysis of novel MLVA-11 *B*. *melitensis* strains isolated in Kuwait. The VNTR copy number derived on the basis of MLVA-11 dataset of each strain is shown corresponding with the lines of the dendrogram. The MLVA-8 and 11 genotypes are mentioned in the columns.

**Fig 4:**
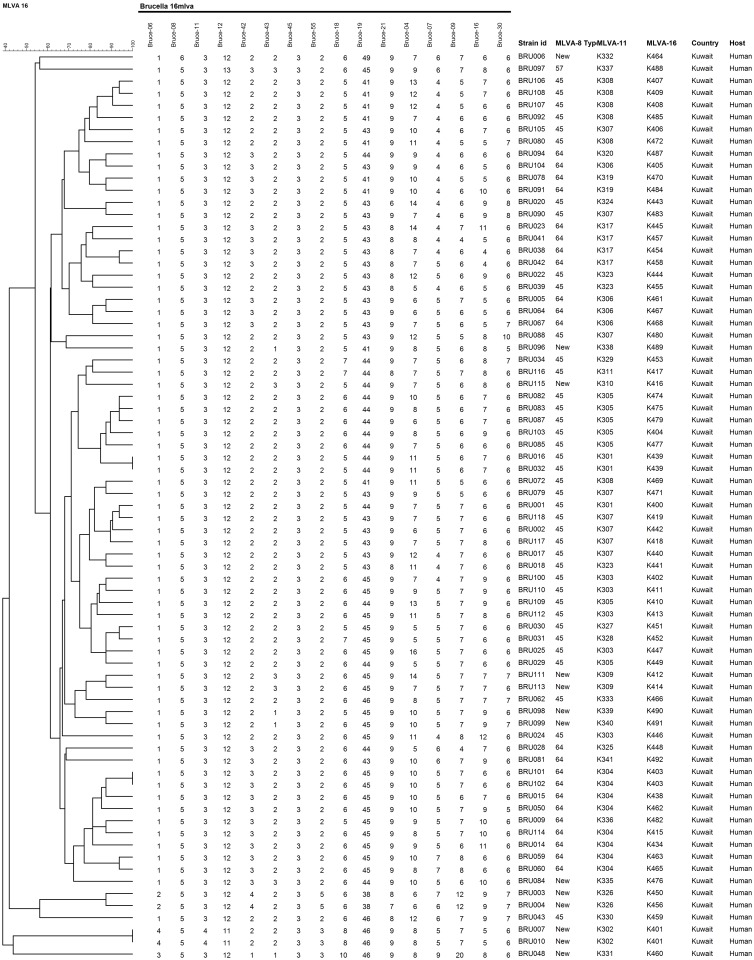
Cluster analysis of *B*. *melitensis* strains based on MLVA-16 assay. The VNTR copy numbers derived on the basis of MLVA-16 dataset of each strain is shown corresponding with the lines of the dendrogram. None of the MLVA-16 genotypes found in the present study were published previously.

Panel 1+2A (MLVA-11) markers separated the ten MLVA-8 types into 32 genotypes, with K304 and K307 bearing the highest numbers of strains (n = 8) (Fig 3). None of the MLVA-11 types have been reported in the MLVA database, and, hence, each MLVA-11 type was given a new designation, from K301 to K341 (Fig 3). MLVA-16 (Panel 1+2A+2B) had the highest discriminating power and further separated forty MLVA-11 types into seventy-two genotypes (Fig 4). The strains BRU007 and BRU010; BRU016 and BRU032; BRU101and BRU102 shared similar MLVA-16 genotypes. The strains BRU007 and BRU010 exhibited novel MLVA-8 genotypes as well (Fig 4).

**Fig 5:**
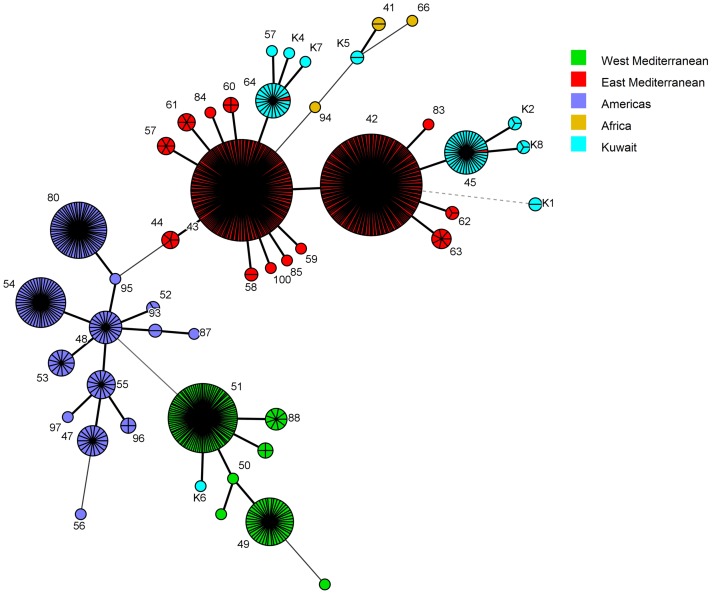
Minimum spanning tree (MST) analysis of published and Kuwaiti *B*. *melitensis* strains using the MLVA-8 data. The published data of 827 strains were downloaded from the MLVA.net database along with the MLVA types of 75 Kuwaiti Strains (S2 Table). The MST was constructed using BioNumerics 7.5 software. The strains were categorized on the basis of their geographic location and differentiated through color codes.

**Fig 6:**
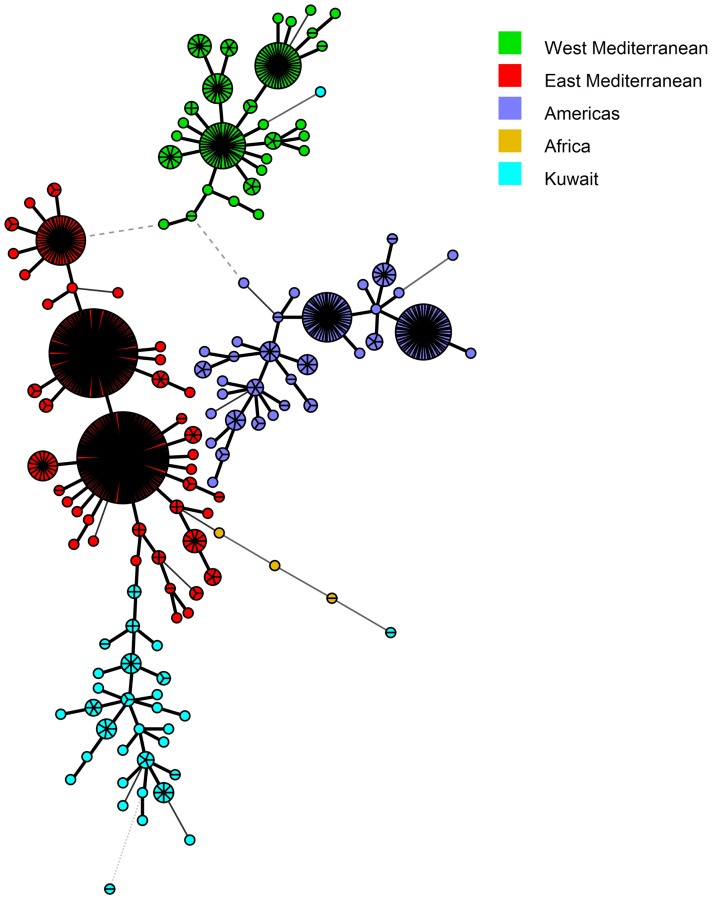
Minimum spanning tree (MST) analysis of published and Kuwaiti *B*. *melitensis* strains using the MLVA-11 data. The published data of 827 strains were downloaded from the MLVA.net database along with the MLVA types of 75 Kuwaiti Strains (S2 Table). The MST was constructed using BioNumerics 7.5 software. The same color codes, as given in Fig 5, were used to differentiate between the strains isolated from Kuwait and other parts of the world.

**Fig 7:**
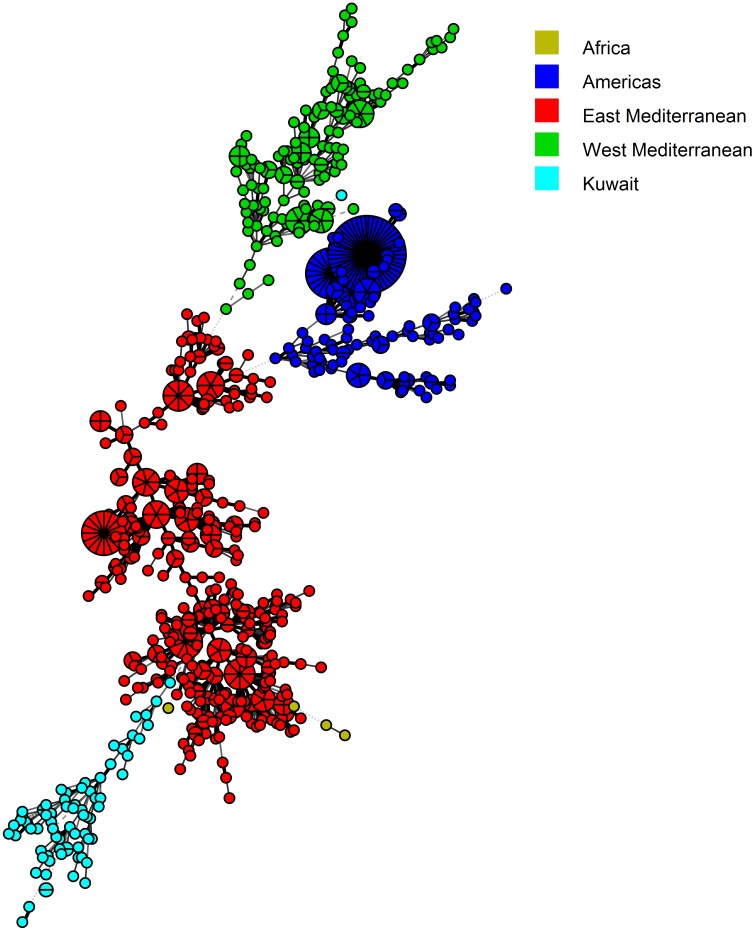
Minimum spanning tree (MST) analysis of published and Kuwaiti *B*. *melitensis* strains using the MLVA-16 data. The published data of 827 strains were downloaded from the MLVA.net database along with the MLVA types of 75 Kuwaiti Strains (S2 Table). The MST was constructed using BioNumerics 7.5 software. The same color codes, as given in Fig 5, were used to differentiate between the strains isolated from Kuwait and other parts of the world.

In order to determine the genetic relatedness of *B*. *melitensis* strains of our study with the strains available in the MLVA database, the VNTR copy numbers of 827 MLVA types from four major clades of the world (Africa, Americas, and East and West Mediterranean Regions) were downloaded from the MLVA database (S2 Table). Neighbor joining minimum spanning trees were constructed from the datasets of the downloaded strains and the strains from Kuwait. This method suggested that the MLVA-8 types found in Kuwait were present as two major groups *i*.*e*. 45 and 64, and the former originated from the latter (Fig 5). Both the groups appeared as a branch in the East Mediterranean clade tree (Fig 5). Interestingly, the genotype 64 showed a minimum distance of 1.0 with MLVA-8 type-43 of a strain isolated in the United Arab Emirates, a country in the East Mediterranean Region [41]. Amongst the new MLVA-8 types, five strains (belonging to K1, K2, K4 and K8) clustered with the East Mediterranean region, two strains (belonging to K5) clustered with the African clade and one strain (belonging to K6) formed a branch in the West Mediterranean Region (Fig 5). In the MST made on the basis of MLVA 11 (panel 1+2A) dataset, a clear cut branching of Type 45 and 64 into several genotypes was observed (Fig 6). The MLVA-16 genotypes appeared as a profusely divided branch of the East Mediterranean clade (Fig 7).

## Discussion

In this study, we have reported the results of testing four molecular methods to identify and genotype *Brucella* spp. infecting humans in Kuwait. The sequencing of 16S rRNA gene suggested that all 75 isolates were *B*. *melitensis*. Sequencing of the 16S rRNA gene is widely used as a speedy and accurate tool for bacterial identification, including *Brucella* [17, 45–47]. The total length of the 16S rRNA gene is ~ 1500 bp, but the bacterial genera and species have been identified based on the first 500 bp region [48]. Suitability of partial 16S rRNA gene sequencing has been demonstrated for identification of dangerous pathogens, including *B*. *melitensis* [48, 49, 50]. The MicroSeq^®^ 500 microbial identification system is a robust and accurate tool for this purpose. It saves time and resources used for full-length gene sequencing. A straight forward workflow allows easy and fast handling and very quick results on the same day. The BioNumerics software aided in maintaining a comprehensive database that could be directly linked to the RDP database and easily accessible Sab and Similarity scores could be obtained rapidly. Although, the inability for intraspecific differentiation of *Brucella* is a limitation of this technique, rapid and accurate identification up to species level is possible through 16S rRNA gene sequencing in a clinical setting.

The real-time PCR and HRM analysis with genus-specific primers identified all the clinical isolates as *Brucella*. Further experiments with species-specific primers confirmed the species as *melitensis*. Winchell *et al*. have reported the method of real-time PCR and HRM analysis for genus and species level identification of this organism [19]. Similar work was carried out recently by Zahidi *et al*. in Malaysia [20]. They concluded that the methodology of real-time PCR combined with HRM analysis was a fast, accurate and cost effective for identification of *B*. *melitensis* [20]. Monitoring the results in real-time saves from additional steps of gel electrophoresis. Moreover, detection of *B*. *melitensis* as a prevalent species in Kuwait would further aid to design eradication strategies, like vaccine development.

The genetic discrimination of *Brucella* remains a challenging task owing to its genetic homogeneity. However, typing of *B*. *melitensis* isolates is highly desirable for contact tracing and epidemiological outbreak investigations in Kuwait and Middle East. ERIC-PCR has been used as a typing tool in the past for *Brucella* [35] and other bacterial species [36]. Our results suggest that ERIC-PCR was a reliable test for identifying genetic differences within *B*. *melitensis* spp. None of our strains shared cent per cent resemblance with each other, hence 75 ERIC genotypes were identified in the region. Owing to the high resolution power of the technique strains sharing only 100% similarity were counted as similar types [36]. The resolution of bands was further improved by the use of Agilent Bioanalyzer. In the present investigation band profiles with high quality sizing resolution (1000–7500 bp: 15%), sizing accuracy (± 10% CV) and sizing reproducibility (5% CV) were obtained, which are essential requirements in fingerprinting based differentiation [51, 52]. In our study, the Agilent Bioanalyzer’s band profiles along with the most advanced version of BioNumerics 7.5 formed a model experiment-analysis combination. The application of DICE algorithm in the BioNumerics software scored the bands as present or absent and created dendrograms that could be used for cluster analysis. The dendrogram of *B*. *melitensis* was classified into two clusters, A and B, based on ~ 80% similarity [36]. The maximum number of strains (n = 51; 68% of total population) were present in the cluster B5. The closeness among 68% of strains of *Brucella* could be attributed to the generation of a large number of identical bands corresponding to the conserved region. However, the typeability was calculated on the basis of limited number of polymorphic bands. Our findings confirm the previous reports of Mercier and co-workers [35] that ERIC-PCR is capable of differentiating between the *Brucella* strains on account of even limited number of polymorphic fragments. In a relatively recent study on the highly homogenous *Corynebacterium pseudotuberculosis*, the ERIC-PCR successfully typed the various strains with high power of discrimination and reproducibility [36].

The species identification and genotyping of all 75 *Brucella* isolates was further extended using the MLVA technique [53–55] and the online *Brucella* database (http://mlva.u-psud.fr/brucella/). This database has been extensively used by other investigators for identification and typing of *Brucella* [27–33, 40–42, 56], and it is regularly updated. The latest version (released on May 16, 2016) contains data for more than 4000 *Brucella* strains of various species. In our study, all isolates were identified as *B*. *melitensis* by the MLVA-8 (Panel 1) typing scheme. However, for the purpose of genotyping, the discriminatory power of the panel 1 (MLVA-8) markers was less as compared to the combined panels 1+2A (MLVA-11) and 1+2A+2B (MLVA-16). All the Panel 2A and 2B primers have different HGDI values in different studies [27, 28, 57, 58]. In our study, Bruce04 (panel 2B marker detecting 11 alleles) exhibited the maximum HGDI value, followed by Bruce16 (panel 2B marker detecting 9 alleles) and Bruce19 (panel 2A marker detecting 7 alleles) (Table 1). The MLVA-16 analysis yielded the maximum number of MLVA genotypes. Similar results have been reported by other investigators [28, 40, 57, 58]. A comparison with strains from other regions revealed considerable variation in the VNTRs associated with the same alleles [27, 29, 41]. Even the strains from same clade exhibit allele differences, except for Bruce 45, which represents a single allele in majority of cases [27, 29, 41].

In order to place our strains in a global perspective, we conducted the MST analysis to establish the genetic relatedness of the genotypes obtained in the current study with MLVA genotypes found worldwide. Based on MLVA-8 analysis, the most common genotypes (45 and 64), identified in the present study, were found in the East Mediterranean clade. Five out of seven novel genotypes also fell into the same clade. One novel genotype each belonged to the African and the West Mediterranean clades. The geographic influence plays a big role in the genotype distribution as none of the other common genotypes of the world were found in Kuwait (Fig 5). Kilic and co-workers reported that the strains isolated in Turkey were mostly from the East Mediterranean region and not from other parts of the world [29]. Interestingly, the genotype 64 was only at a distance of 1.0 with the genotype 43, which is commonly found in Turkey [29] and UAE [41]. Genotype 45 was also found previously in Turkey [39] and China [57]. Hence we assume that the *Brucella* have probably entered in Kuwait through animals and livestock imported from nearby regions. The strains isolated from Turkey [29], UAE [41], Lebanon [28] and China [57] also had genotypes belonging to the East Mediterranean region. The MLVA genotypes of Kuwait form a distinct branch in the East Mediterranean region with two separate groups. The process of evolution may have resulted in the formation of new genotypes. Further investigations on the novel genotype associated with the East Mediterranean region should be done. Owing to the further differentiation on the basis of panel 1+2a and Panel 1+2a+2b, the Kuwaiti arm of the East Mediterranean region forms a branched tree suggesting the presence of diverse genotypes of *B*. *melitensis* in the region.

In conclusion, our work demonstrates that the molecular techniques are fast and accurate tools for identifying and discriminating the strains of *Brucella* in Kuwait. The region is dominated by the pathogenic species of *B*. *melitensis*. 16S rRNA gene sequencing using MicroSeq^®^ 500 kit and real-time PCR can provide rapid confirmatory identification of *Brucella* isolates up to species level. The ERIC-PCR has a higher discriminatory power and a potent tool for intra-species diversification. The technique suggests the presence of 75 ERIC genotypes in Kuwait. However, the ERIC-PCR is quite limited due to non-availability of comparative data set from other studies. The MLVA-16 genotyping scheme is capable of identifying the isolates up to species and genotype levels as well as trace back the origin of strains in a particular region. The strains in Kuwait have their origin from the East Mediterranean region and are in close resemblance with UAE strains.

## Supporting information

S1 Fig A Bioanlayzer gel image showing PCR amplification of the 16S rRNA gene by using the MicroSeq kit. Ladder: represents the Agilent DNA 1000 bp Ladder (numbers correspond to the base pairs of each fragment), PC: Positive control (*E*. *coli* DNA supplied along with the kit), NC: Negative control (water, shows no amplification), Lanes 100, 1, 3 and 38 represent clinical strains BRU100, BRU001, BRU003 and BRU038 used in this study, respectively. The PC and clinical strains show the amplification of a band at about 500 bp.
(TIFF)

S2 Fig A Bioanalyzer gel image showing the ERIC fingerprints of random strains of *Brucella*. DNA-7500 (50–10,000 bp) was used as the size standard shown in the lane marked as ladder. The remaining lanes are for representative strains chosen for ERIC analysis.
(TIFF)

S3 Fig Agarose gel (0.8%) showing ERIC profiles of three technical replicates done on two consecutive days. (a) three replicates of BRU 001 ran on day 1; (b) three replicates of BRU001 ran after the second PCR on day 2; (c) three replicates of BRU003 ran on day1; (d) three replicates of BRU003 ran after the second PCR on day 2; L is the ladder (100bp + 1KB mixed)
(TIFF)

S1 Table The nucleotide lengths of the PCR products obtained after 25 cycles of 16S rDNA sequencing PCR. (XLSX)

S2 Table The VNTR copy numbers of 827 MLVA types from four major clades of the world (Africa, America, East and West Mediterranean regions) downloaded from the MLVA database and 75 Kuwaiti isolates (numbers 828 to 902). (XLSX)

S1 File A representative experiment showing Bioanalyzer analysis for DNA band patterns and electropherograms using PCR products of MLVA primers. (PDF)
