# Supplementary material for: Species identification and molecular typing of human Brucella isolates from Kuwait
Source: PLoS One. 2017 Aug 11;12(8):e0182111. doi: 10.1371/journal.pone.0182111 (PMC5553756; doi:10.1371/journal.pone.0182111)
Supplement: S1 File — (PDF) [file pone.0182111.s006.pdf]

Assay Class: DNA 1000
Data Path: C:\...-16\2100 expert_DNA 1000_DE72901399_2015-11-16_09-53-21.xad

Created: 11/16/2015 9:53:20 AM
Modified: 2/2/2016 8:53:35 AM

## Electrophoresis File Run Summary

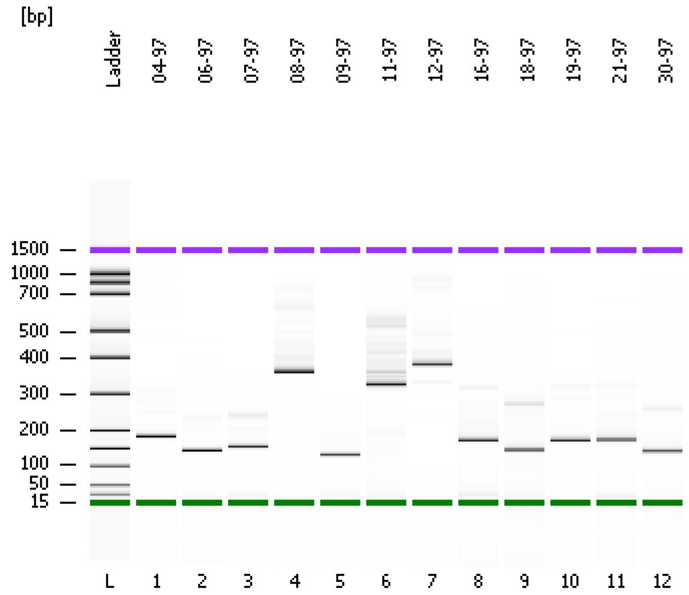


Instrument Information:

| | | | |
|---|---|---|---|
| Instrument Name: | DE72901399 | Firmware: | C.01.069 |
| Serial#: | DE72901399 | Type: | G2939A |

Assay Information:

| | |
|---|---|
| Assay Origin Path: | C:\Program Files\Agilent\2100 bioanalyzer\2100 expert\assays\dsDNA\DNA 1000 Series II.xsy |
| Assay Class: | DNA 1000 |
| Version: | 2.3 |
| Assay Comments: | DNA Analysis 25 -1000 bp |

© Copyright 2003-2009 Agilent Technologies, Inc.

Chip Information:

Chip Lot #:

Reagent Kit Lot #:

Chip Comments:

**04-97**

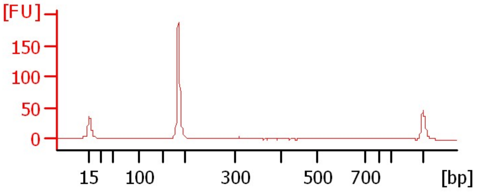


**06-97**

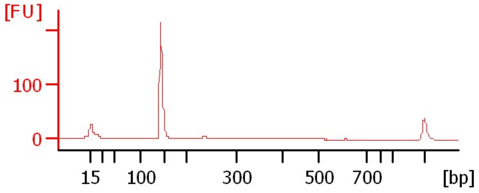


**07-97**

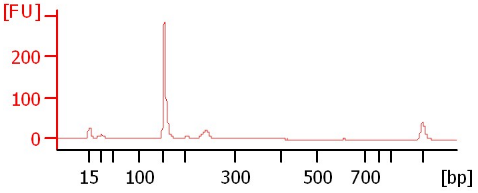


**08-97**

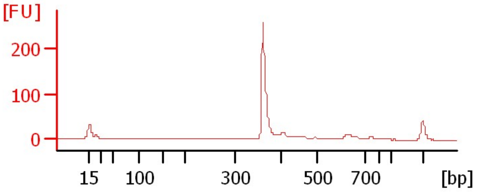


**09-97**

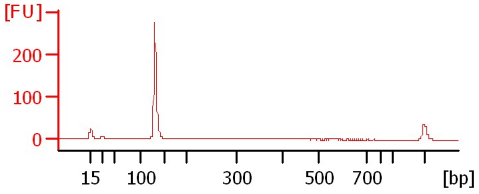


**11-97**

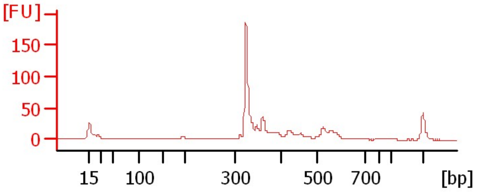


**12-97**

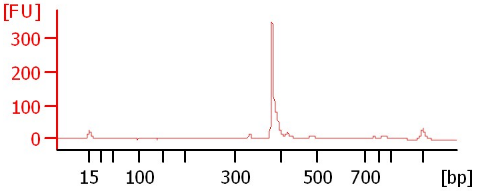


**16-97**

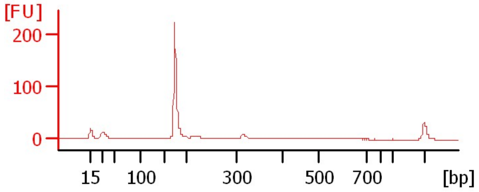


**18-97**

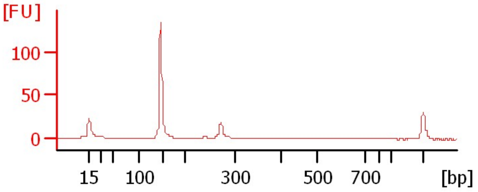


**19-97**

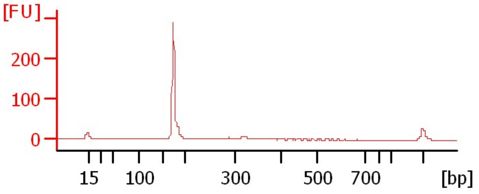


**21-97**

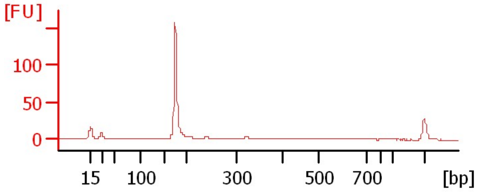


**30-97**

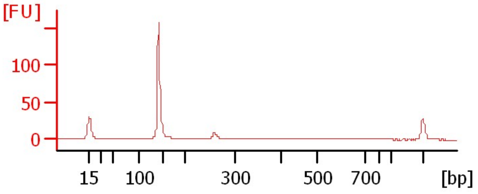

Assay Class: DNA 1000 Created: 11/16/2015 9:53:20 AM
Data Path: C:\...-16\2100 expert_DNA 1000_DE72901399_2015-11-16_09-53-21.xad Modified: 2/2/2016 8:53:35 AM

## Electrophoresis File Run Summary (Chip Summary)

| Sample Name | Sample Comment | Rest. Digest | Status | Observation | Result Label | Result Color |
|---|---|---|---|---|---|---|
| 04-97 | | ☐ | ✓ | | | |
| 06-97 | | ☐ | ✓ | | | |
| 07-97 | | ☐ | ✓ | | | |
| 08-97 | | ☐ | ✓ | | | |
| 09-97 | | ☐ | ✓ | | | |
| 11-97 | | ☐ | ✓ | | | |
| 12-97 | | ☐ | ✓ | | | |
| 16-97 | | ☐ | ✓ | | | |
| 18-97 | | ☐ | ✓ | | | |
| 19-97 | | ☐ | ✓ | | | |
| 21-97 | | ☐ | ✓ | | | |
| 30-97 | | ☐ | ✓ | | | |
| Ladder | | ☐ | ✓ | | | |

**Chip Lot #** **Reagent Kit Lot #**

**Chip Comments :**

 © Copyright 2003 - 2009 Agilent Technologies, Inc. 

Assay Class: DNA 1000 Created: 11/16/2015 9:53:20 AM
Data Path: C:\...-16\2100 expert_DNA 1000_DE72901399_2015-11-16_09-53-21.xad Modified: 2/2/2016 8:53:35 AM

## Electrophoresis Assay Details

### General Analysis Settings

Number of Available Sample and Ladder Wells (Max.) : 13
Minimum Visible Range [s] : 30
Maximum Visible Range [s] : 129
Start Analysis Time Range [s] : 30
End Analysis Time Range [s] : 128.95
Ladder Concentration [ng/µl] : 44
Uses Standard Area for Ladder Fragments
Lower Marker Concentration [ng/µl] : 4.2
Upper Marker Concentration [ng/µl] : 2.1
Used Upper Marker for Quantitation
Standard Curve Fit is Point to Point
Show Data Aligned to Lower and Upper Marker

### Integrator Settings

Integration Start Time [s] : 30
Integration End Time [s] : 128.95
Slope Threshold : 0.5
Height Threshold [FU] : 20
Area Threshold : 0.1
Width Threshold [s] : 0.5
Baseline Plateau [s] : 0.5

### Filter Settings

Filter Width [s] : 0.5
Polynomial Order : 4

### Ladder

| Ladder Peak | Size | Area |
|---|---|---|
| 1 | 15 | 25 |
| 2 | 25 | 26 |
| 3 | 50 | 34 |
| 4 | 100 | 41 |
| 5 | 150 | 45 |
| 6 | 200 | 52 |
| 7 | 300 | 63 |
| 8 | 400 | 76 |
| 9 | 500 | 83 |
| 10 | 700 | 88 |
| 11 | 850 | 86 |
| 12 | 1000 | 90 |
| 13 | 1500 | 52 |

 © Copyright 2003 - 2009 Agilent Technologies, Inc. 

Assay Class: DNA 1000
Data Path: C:\...-16\2100 expert_DNA 1000_DE72901399_2015-11-16_09-53-21.xad

Created: 11/16/2015 9:53:20 AM
Modified: 2/2/2016 8:53:35 AM

**Electropherogram Summary**

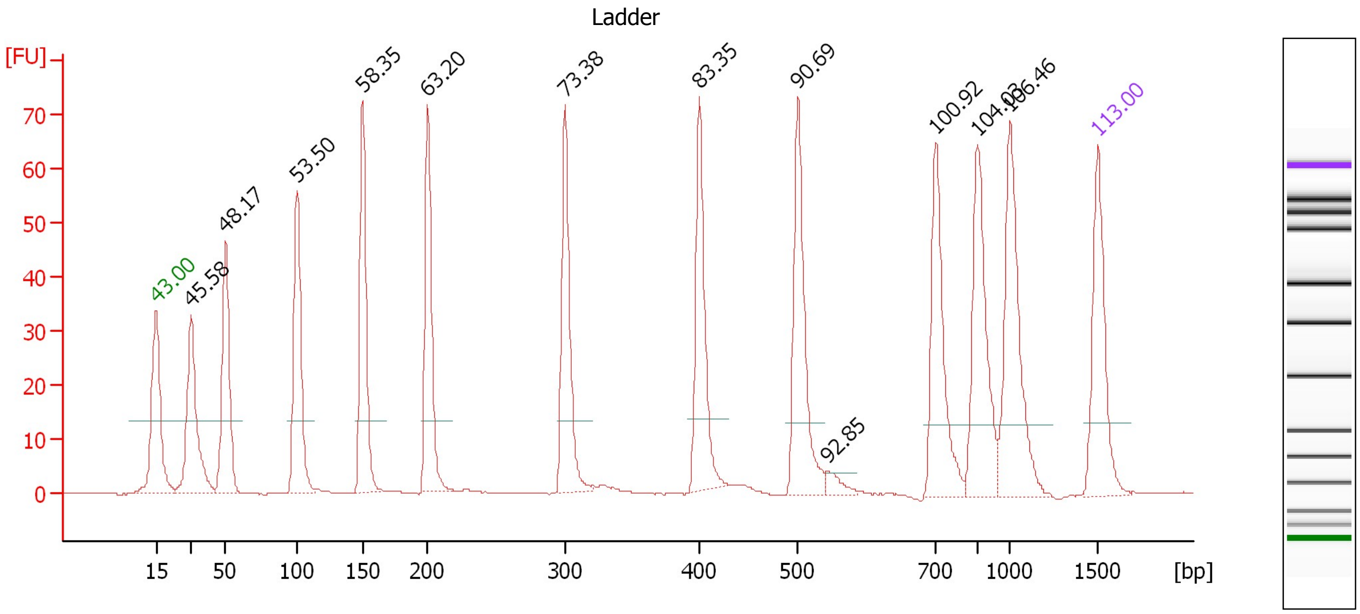


**Peak table for Ladder**

| Peak | | Size [bp] | Conc. [ng/µl] | Molarity [nmol/l] | Observations |
|---|---|---|---|---|---|
| 1 | ◀ | 15 | 4.20 | 424.2 | Lower Marker |
| 2 | L | 25 | 4.00 | 242.4 | Ladder Peak |
| 3 | L | 50 | 4.00 | 121.2 | Ladder Peak |
| 4 | L | 100 | 4.00 | 60.6 | Ladder Peak |
| 5 | L | 150 | 4.00 | 40.4 | Ladder Peak |
| 6 | L | 200 | 4.00 | 30.3 | Ladder Peak |
| 7 | L | 300 | 4.00 | 20.2 | Ladder Peak |
| 8 | L | 400 | 4.00 | 15.2 | Ladder Peak |
| 9 | L | 500 | 4.00 | 12.1 | Ladder Peak |
| 10 | | 542 | 0.00 | 0.0 | |
| 11 | L | 700 | 4.00 | 8.7 | Ladder Peak |
| 12 | L | 850 | 4.00 | 7.1 | Ladder Peak |
| 13 | L | 1,000 | 4.00 | 6.1 | Ladder Peak |
| 14 | ▶ | 1,500 | 2.10 | 2.1 | Upper Marker |

 © Copyright 2003 - 2009 Agilent Technologies, Inc. 

Assay Class: DNA 1000
Data Path: C:\...-16\2100 expert_DNA 1000_DE72901399_2015-11-16_09-53-21.xad
Created: 11/16/2015 9:53:20 AM
Modified: 2/2/2016 8:53:35 AM

**Electropherogram Summary Continued ...**

04-97

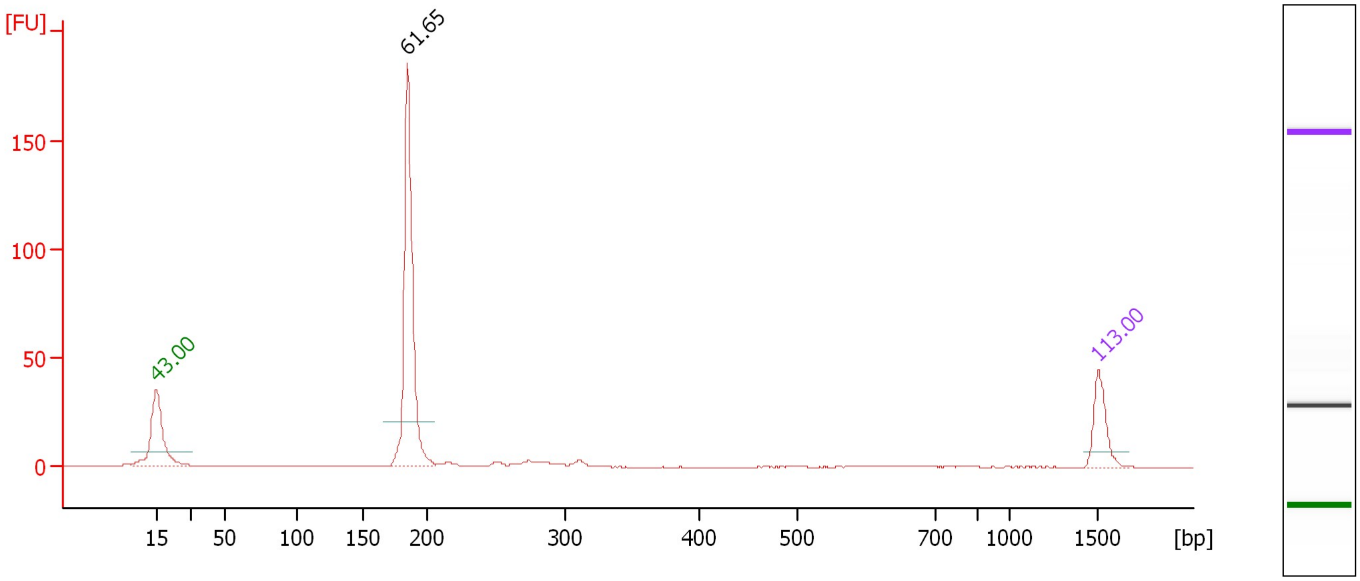


**Overall Results for sample 1 : 04-97**

Number of peaks found: 1

**Peak table for sample 1 : 04-97**

| Peak | | Size [bp] | Conc. [ng/µl] | Molarity [nmol/l] | Observations |
|---|---|---|---|---|---|
| 1 | ◀ | 15 | 4.20 | 424.2 | Lower Marker |
| 2 | | 184 | 11.71 | 96.4 | |
| 3 | ▶ | 1,500 | 2.10 | 2.1 | Upper Marker |

 © Copyright 2003 - 2009 Agilent Technologies, Inc. 

Assay Class: DNA 1000
Data Path: C:\...-16\2100 expert_DNA 1000_DE72901399_2015-11-16_09-53-21.xad

Created: 11/16/2015 9:53:20 AM
Modified: 2/2/2016 8:53:35 AM

**Electropherogram Summary Continued ...**

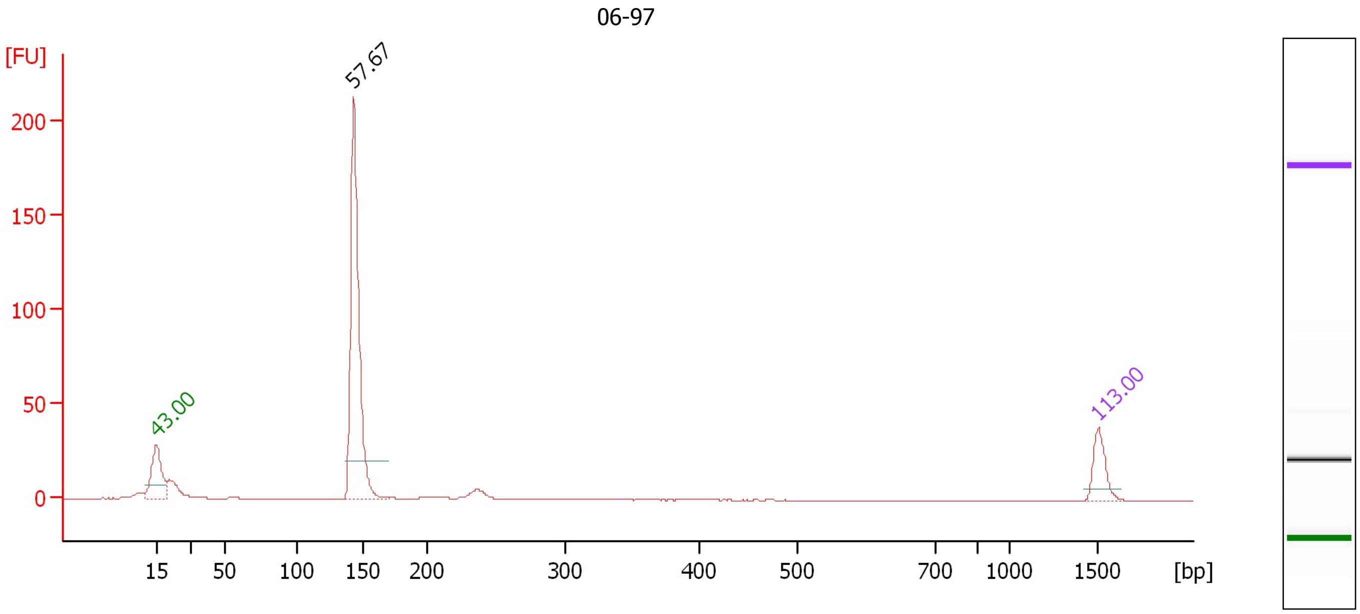


**Overall Results for sample 2 : 06-97**

Number of peaks found: 1

**Peak table for sample 2 : 06-97**

| Peak | | Size [bp] | Conc. [ng/µl] | Molarity [nmol/l] | Observations |
|---|---|---|---|---|---|
| 1 | ◀ | 15 | 4.20 | 424.2 | Lower Marker |
| 2 | | 143 | 17.37 | 184.0 | |
| 3 | ▶ | 1,500 | 2.10 | 2.1 | Upper Marker |

| | | | |
|---|---|---|---|
| Assay Class: | DNA 1000 | Created: | 11/16/2015 9:53:20 AM |
| Data Path: | C:\...-16\2100 expert_DNA 1000_DE72901399_2015-11-16_09-53-21.xad | Modified: | 2/2/2016 8:53:35 AM |

**Electropherogram Summary Continued ...**

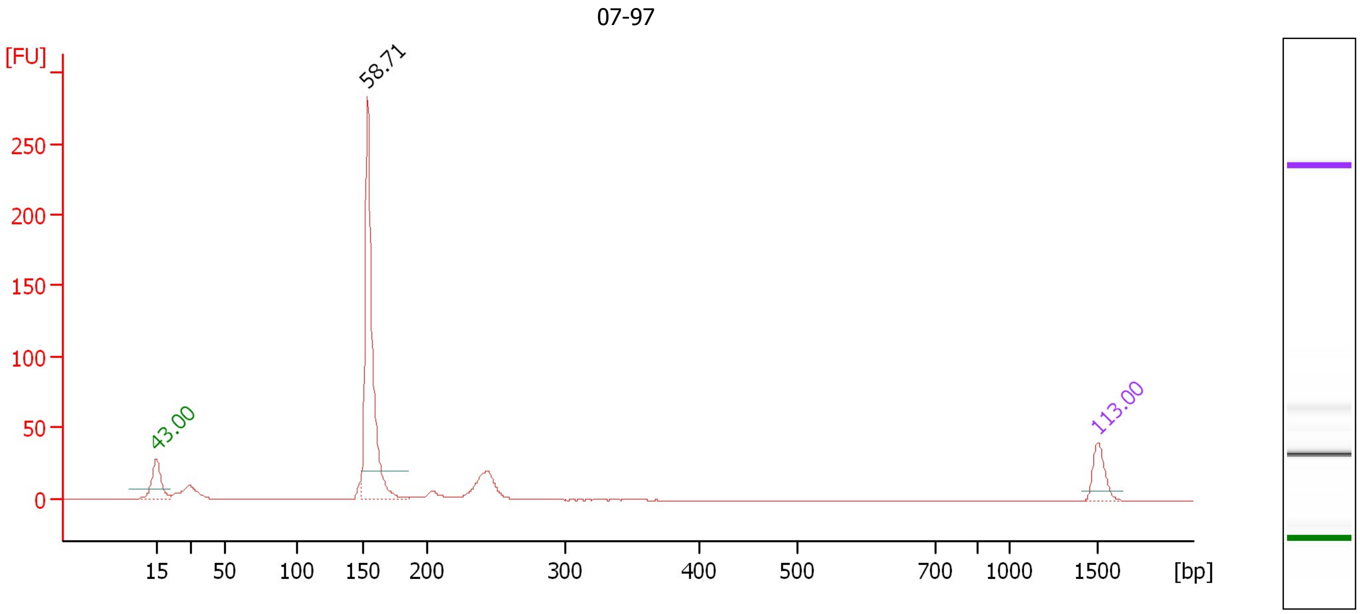


**Overall Results for sample 3 :** **07-97**

Number of peaks found: 1

**Peak table for sample 3 :** **07-97**

| Peak | | Size [bp] | Conc. [ng/µl] | Molarity [nmol/l] | Observations |
|---|---|---|---|---|---|
| 1 | ◀ | 15 | 4.20 | 424.2 | Lower Marker |
| 2 | | 154 | 18.84 | 185.6 | |
| 3 | ▶ | 1,500 | 2.10 | 2.1 | Upper Marker |

 © Copyright 2003 - 2009 Agilent Technologies, Inc. 

| | | | |
|---|---|---|---|
| Assay Class: | DNA 1000 | Created: | 11/16/2015 9:53:20 AM |
| Data Path: | C:\...-16\2100 expert_DNA 1000_DE72901399_2015-11-16_09-53-21.xad | Modified: | 2/2/2016 8:53:35 AM |

**Electropherogram Summary Continued ...**

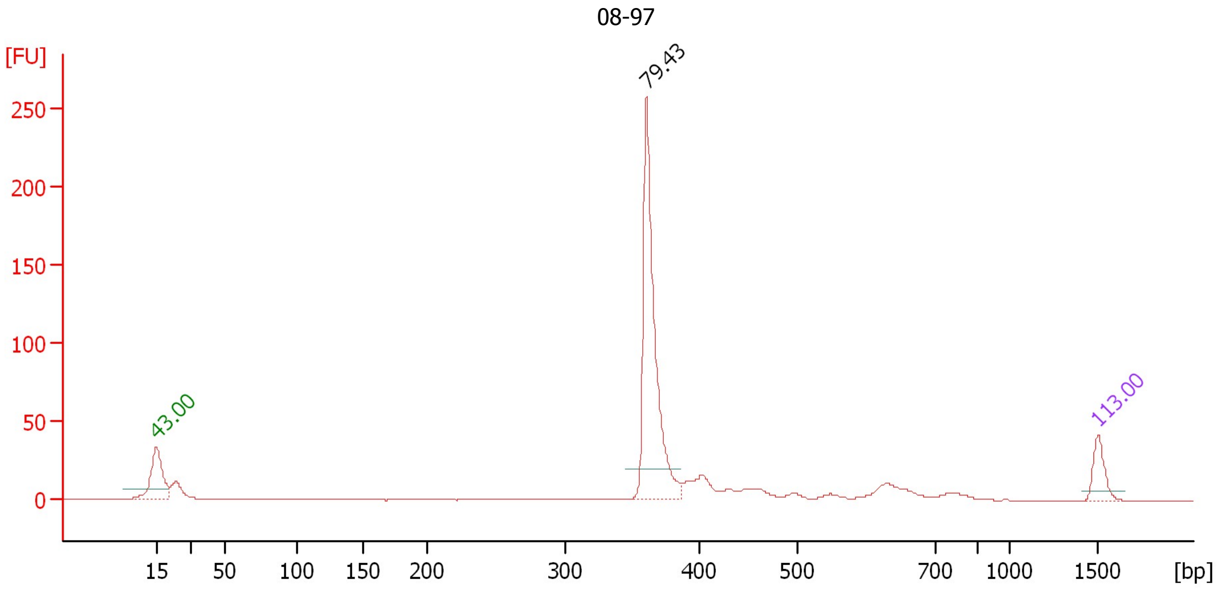


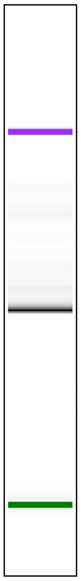

**Overall Results for sample 4 : 08-97**

Number of peaks found: 1

**Peak table for sample 4 : 08-97**

| Peak | | Size [bp] | Conc. [ng/µl] | Molarity [nmol/l] | Observations |
|---|---|---|---|---|---|
| 1 | ◀ | 15 | 4.20 | 424.2 | Lower Marker |
| 2 | | 361 | 17.68 | 74.3 | |
| 3 | ▶ | 1,500 | 2.10 | 2.1 | Upper Marker |

Assay Class: DNA 1000 Created: 11/16/2015 9:53:20 AM
Data Path: C:\...-16\2100 expert_DNA 1000_DE72901399_2015-11-16_09-53-21.xad Modified: 2/2/2016 8:53:35 AM

**Electropherogram Summary Continued ...**

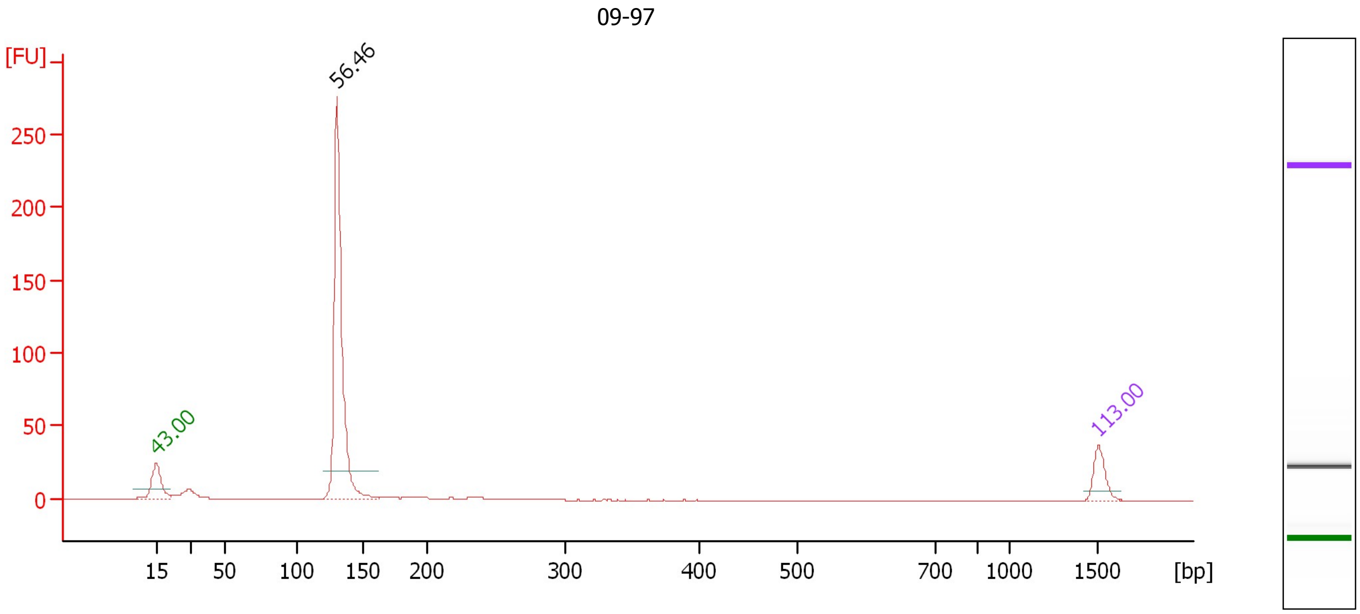


**Overall Results for sample 5 : 09-97**

Number of peaks found: 1

**Peak table for sample 5 : 09-97**

| Peak | | Size [bp] | Conc. [ng/µl] | Molarity [nmol/l] | Observations |
|---|---|---|---|---|---|
| 1 | ◀ | 15 | 4.20 | 424.2 | Lower Marker |
| 2 | | 131 | 22.35 | 259.4 | |
| 3 | ▶ | 1,500 | 2.10 | 2.1 | Upper Marker |

 © Copyright 2003 - 2009 Agilent Technologies, Inc. 

Assay Class: DNA 1000
Data Path: C:\...-16\2100 expert_DNA 1000_DE72901399_2015-11-16_09-53-21.xad

Created: 11/16/2015 9:53:20 AM
Modified: 2/2/2016 8:53:35 AM

**Electropherogram Summary Continued ...**

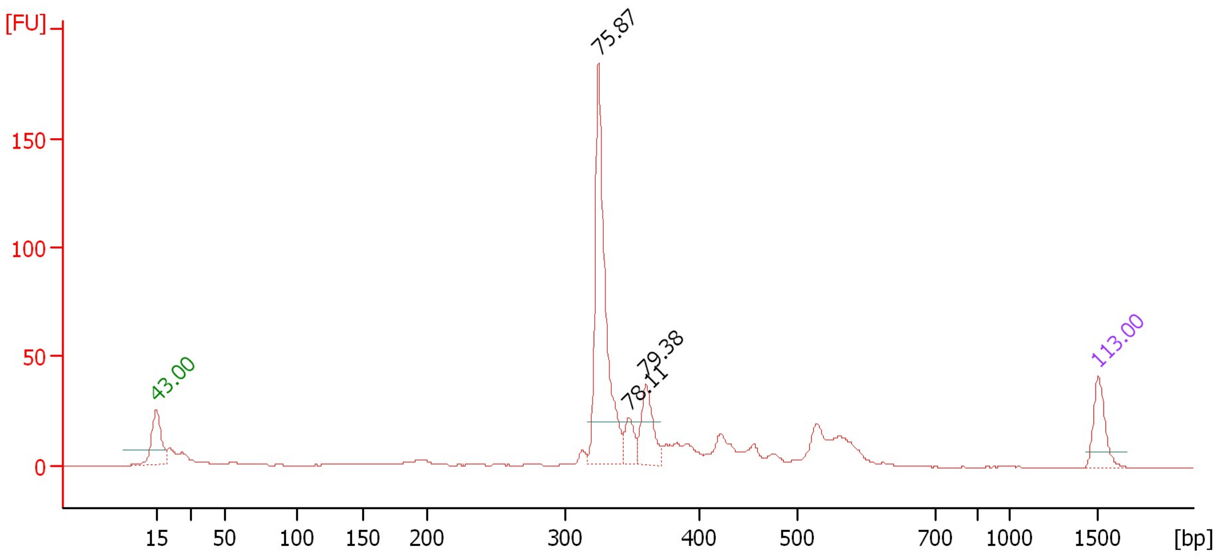


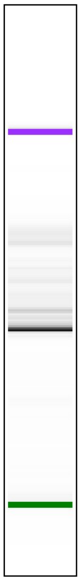

**Overall Results for sample 6 : 11-97**

Number of peaks found: 3

**Peak table for sample 6 : 11-97**

| Peak | | Size [bp] | Conc. [ng/µl] | Molarity [nmol/l] | Observations |
|---|---|---|---|---|---|
| 1 | ◀ | 15 | 4.20 | 424.2 | Lower Marker |
| 2 | | 325 | 12.39 | 57.8 | |
| 3 | | 347 | 1.33 | 5.8 | |
| 4 | | 360 | 2.74 | 11.5 | |
| 5 | ▶ | 1,500 | 2.10 | 2.1 | Upper Marker |

 © Copyright 2003 - 2009 Agilent Technologies, Inc. 

Assay Class: DNA 1000
Data Path: C:\...-16\2100 expert_DNA 1000_DE72901399_2015-11-16_09-53-21.xad

Created: 11/16/2015 9:53:20 AM
Modified: 2/2/2016 8:53:35 AM

**Electropherogram Summary Continued ...**

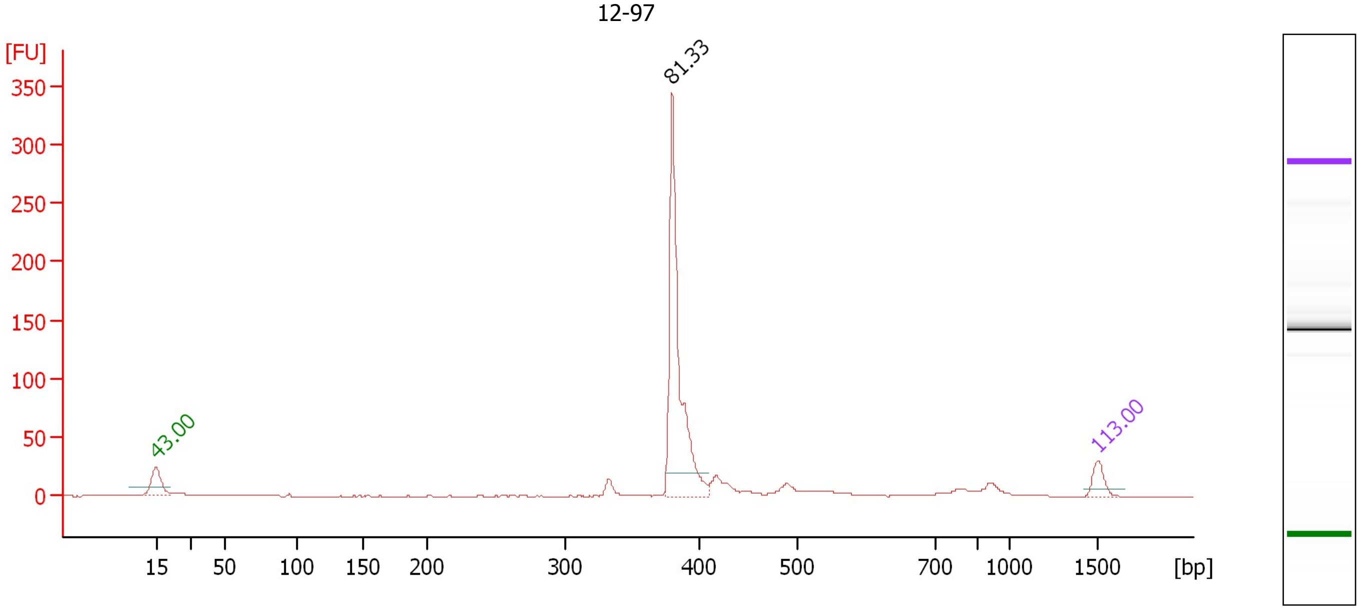


**Overall Results for sample 7 : 12-97**

Number of peaks found: 1

**Peak table for sample 7 : 12-97**

| Peak | | Size [bp] | Conc. [ng/µl] | Molarity [nmol/l] | Observations |
|---|---|---|---|---|---|
| 1 | ◀ | 15 | 4.20 | 424.2 | Lower Marker |
| 2 | | 380 | 26.78 | 106.9 | |
| 3 | ▶ | 1,500 | 2.10 | 2.1 | Upper Marker |

 © Copyright 2003 - 2009 Agilent Technologies, Inc. 

Assay Class: DNA 1000 | Created: 11/16/2015 9:53:20 AM
Data Path: C:\...-16\2100 expert_DNA 1000_DE72901399_2015-11-16_09-53-21.xad | Modified: 2/2/2016 8:53:35 AM

## Electropherogram Summary Continued ...

16-97

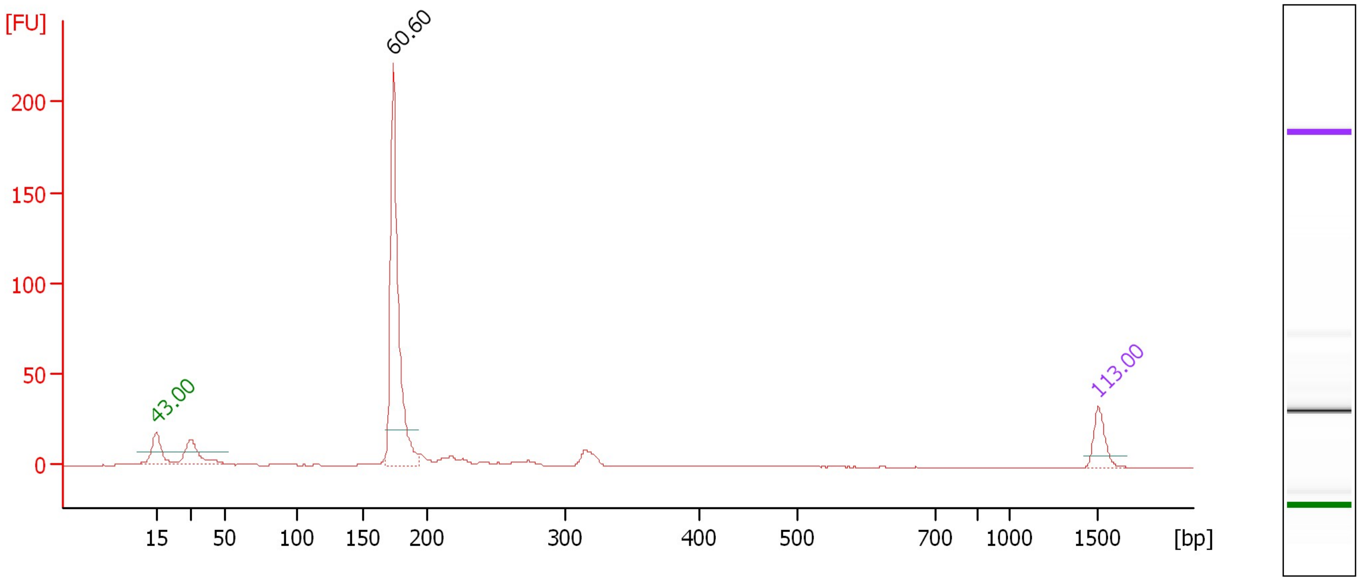


**Overall Results for sample 8 :** **16-97**

Number of peaks found: 1

**Peak table for sample 8 :** **16-97**

| Peak | | Size [bp] | Conc. [ng/µl] | Molarity [nmol/l] | Observations |
|---|---|---|---|---|---|
| 1 | ◀ | 15 | 4.20 | 424.2 | Lower Marker |
| 2 | | 173 | 19.57 | 171.3 | |
| 3 | ▶ | 1,500 | 2.10 | 2.1 | Upper Marker |

 © Copyright 2003 - 2009 Agilent Technologies, Inc. 

Assay Class: DNA 1000
Data Path: C:\...-16\2100 expert_DNA 1000_DE72901399_2015-11-16_09-53-21.xad
Created: 11/16/2015 9:53:20 AM
Modified: 2/2/2016 8:53:35 AM

**Electropherogram Summary Continued ...**

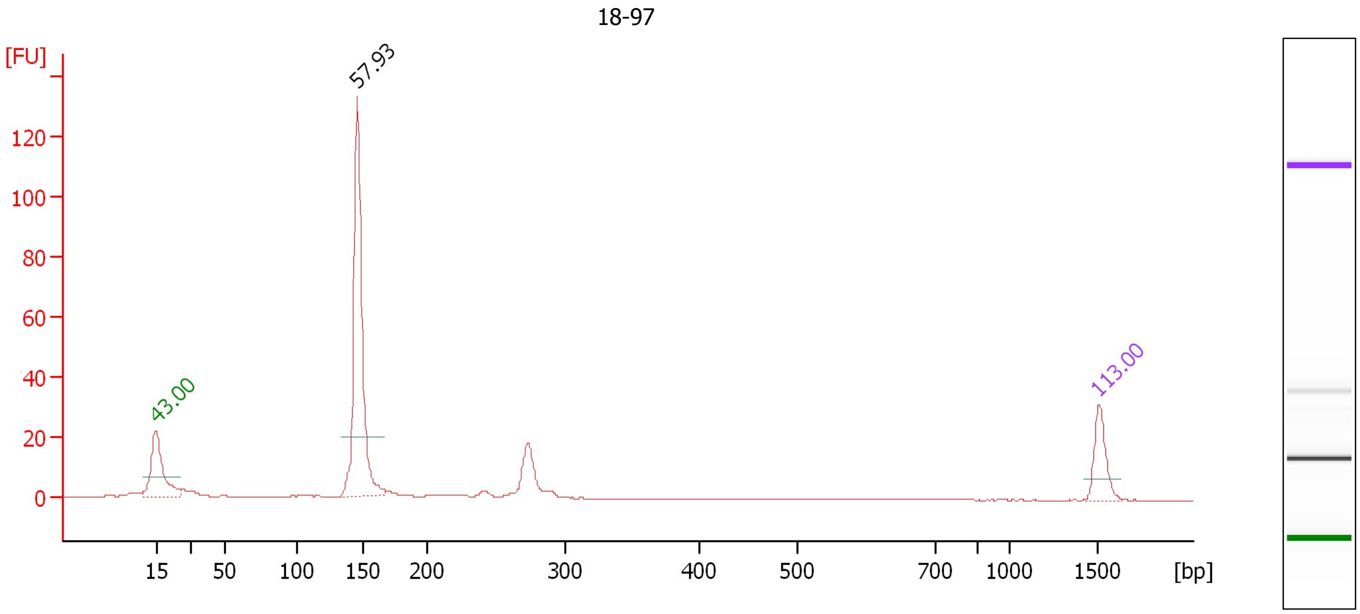


**Overall Results for sample 9 : 18-97**

Number of peaks found: 1

**Peak table for sample 9 : 18-97**

| Peak | | Size [bp] | Conc. [ng/µl] | Molarity [nmol/l] | Observations |
|---|---|---|---|---|---|
| 1 | ◀ | 15 | 4.20 | 424.2 | Lower Marker |
| 2 | | 146 | 13.69 | 142.3 | |
| 3 | ▶ | 1,500 | 2.10 | 2.1 | Upper Marker |

 © Copyright 2003 - 2009 Agilent Technologies, Inc. 

Assay Class: DNA 1000 Created: 11/16/2015 9:53:20 AM
Data Path: C:\...-16\2100 expert_DNA 1000_DE72901399_2015-11-16_09-53-21.xad Modified: 2/2/2016 8:53:35 AM

**Electropherogram Summary Continued ...**

19-97

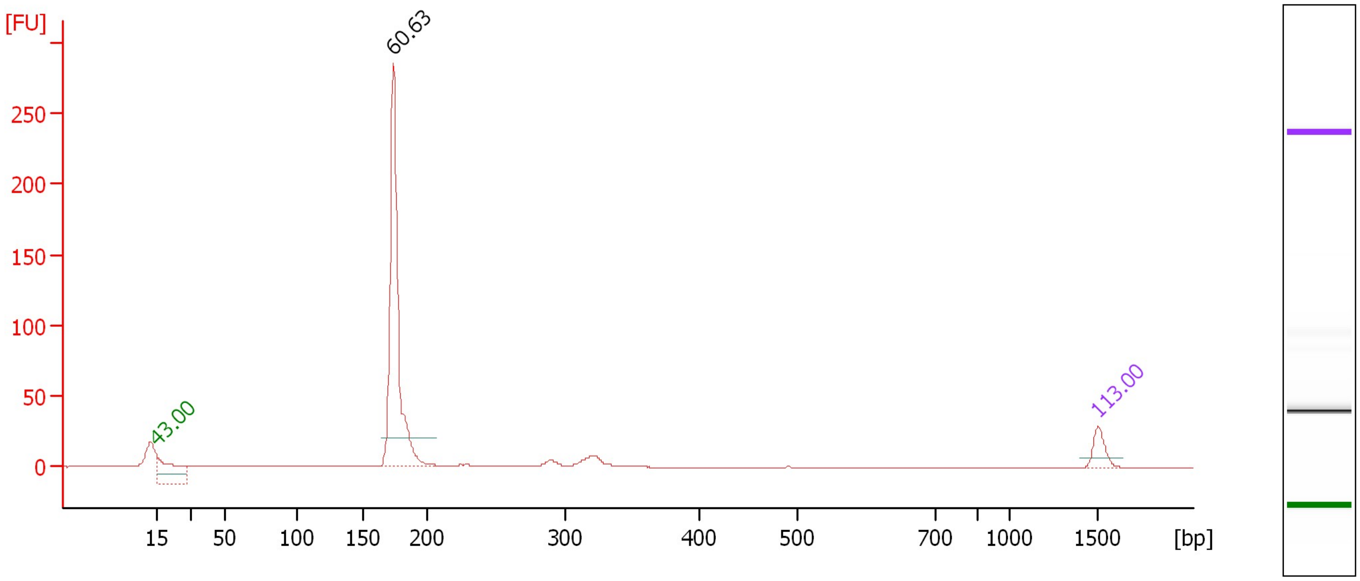


**Overall Results for sample 10 : 19-97**

Number of peaks found: 1

**Peak table for sample 10 : 19-97**

| Peak | | Size [bp] | Conc. [ng/µl] | Molarity [nmol/l] | Observations |
|---|---|---|---|---|---|
| 1 | ◀ | 15 | 4.20 | 424.2 | Lower Marker |
| 2 | | 173 | 28.60 | 249.8 | |
| 3 | ▶ | 1,500 | 2.10 | 2.1 | Upper Marker |

| | | | |
|---|---|---|---|
| Assay Class: | DNA 1000 | Created: | 11/16/2015 9:53:20 AM |
| Data Path: | C:\...-16\2100 expert_DNA 1000_DE72901399_2015-11-16_09-53-21.xad | Modified: | 2/2/2016 8:53:35 AM |

**Electropherogram Summary Continued ...**

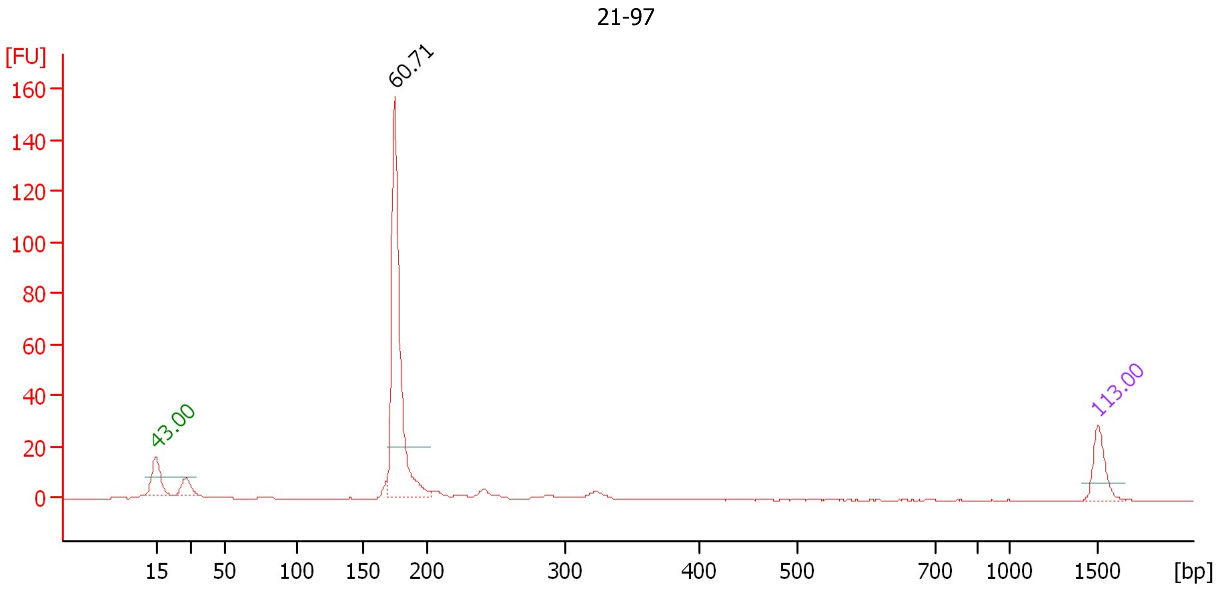


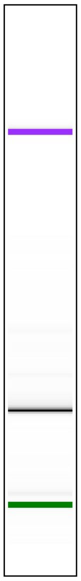

**Overall Results for sample 11 : 21-97**

Number of peaks found: 1

**Peak table for sample 11 : 21-97**

| Peak | | Size [bp] | Conc. [ng/µl] | Molarity [nmol/l] | Observations |
|---|---|---|---|---|---|
| 1 | ◀ | 15 | 4.20 | 424.2 | Lower Marker |
| 2 | | 174 | 16.34 | 142.1 | |
| 3 | ▶ | 1,500 | 2.10 | 2.1 | Upper Marker |

 © Copyright 2003 - 2009 Agilent Technologies, Inc. 

Assay Class: DNA 1000 Created: 11/16/2015 9:53:20 AM
Data Path: C:\...-16\2100 expert_DNA 1000_DE72901399_2015-11-16_09-53-21.xad Modified: 2/2/2016 8:53:35 AM

**Electropherogram Summary Continued ...**

30-97

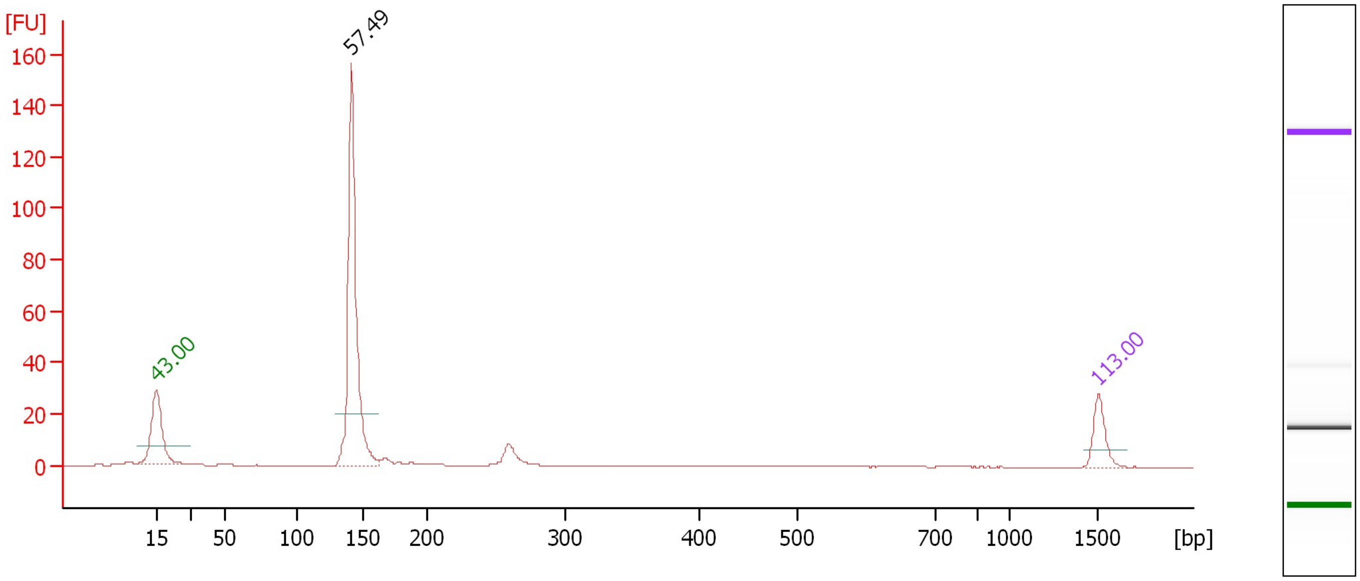


**Overall Results for sample 12 : 30-97**

Number of peaks found: 1

**Peak table for sample 12 : 30-97**

| Peak | | Size [bp] | Conc. [ng/µl] | Molarity [nmol/l] | Observations |
|---|---|---|---|---|---|
| 1 | ◀ | 15 | 4.20 | 424.2 | Lower Marker |
| 2 | | 141 | 17.75 | 190.4 | |
| 3 | ▶ | 1,500 | 2.10 | 2.1 | Upper Marker |

© Copyright 2003 - 2009 Agilent Technologies, Inc.

Assay Class: DNA 1000
Data Path: C:\...-16\2100 expert_DNA 1000_DE72901399_2015-11-16_09-53-21.xad

Created: 11/16/2015 9:53:20 AM
Modified: 2/2/2016 8:53:35 AM

**Gel Image**

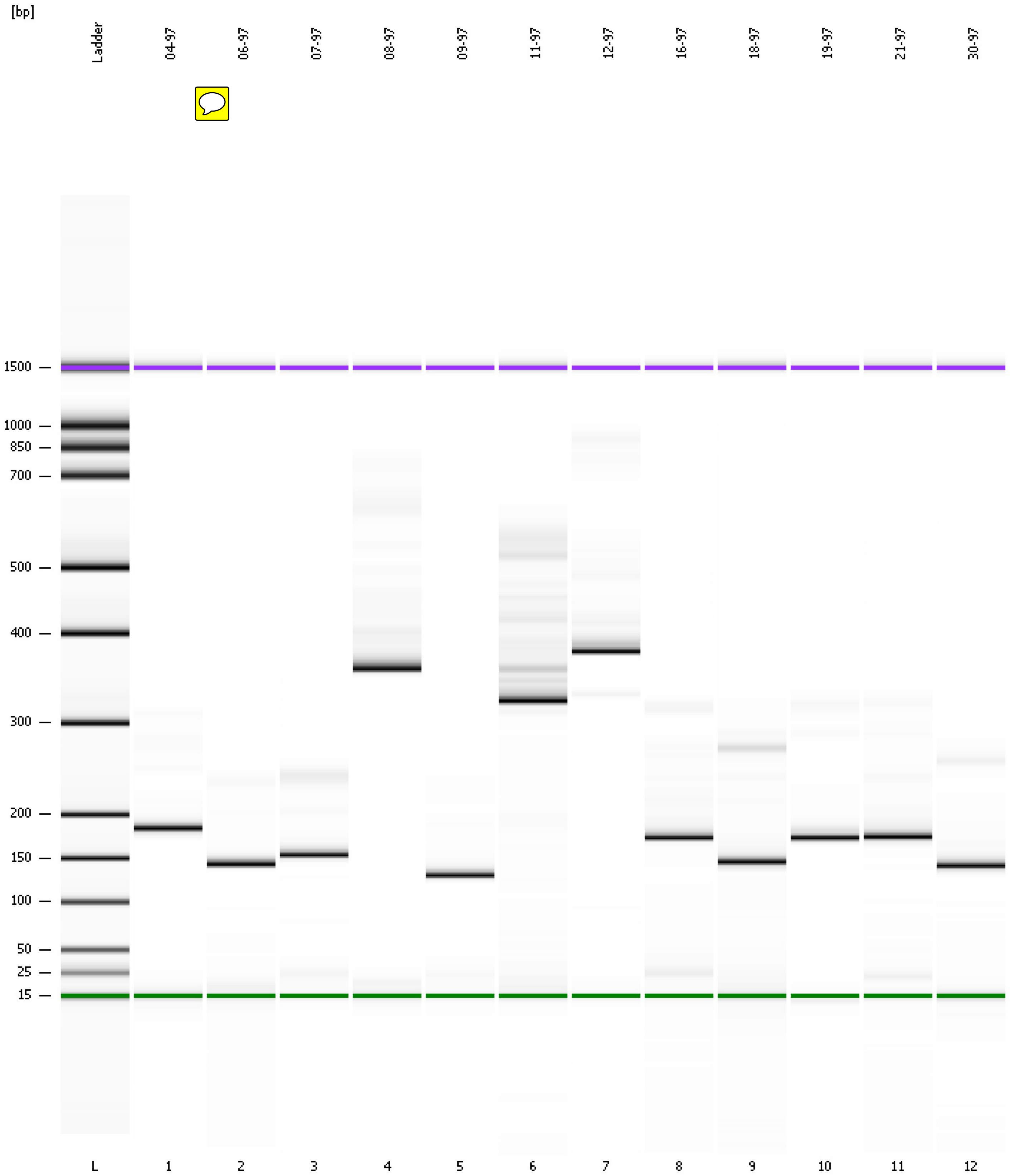


 © Copyright 2003 - 2009 Agilent Technologies, Inc. 

Assay Class: DNA 1000
Data Path: C:\...-16\2100 expert_DNA 1000_DE72901399_2015-11-16_09-53-21.xad

Created: 11/16/2015 9:53:20 AM
Modified: 2/2/2016 8:53:35 AM

## Run Logbook

| Description | Number | Source | Category | Sub Category | Time | Time Zone | User | Host |
|---|---|---|---|---|---|---|---|---|
| Run ended on port 2 (Number of wells acquired: 13) | | Instrument | Run | | 11/16/2015 10:35:12 AM | (GMT +01:00) W. Europe Standard Time | Administrator | V-RCF-G1-AG2100 |
| Run started on port 2 (File: C:\Program Files\Agilent\2100 bioanalyzer\2100 expert\Data\2015-11-16\2100 expert_DNA 1000_DE72901399_2015-11-16_09-53-21.xad) | | Instrument | Run | | 11/16/2015 9:53:27 AM | (GMT +01:00) W. Europe Standard Time | Administrator | V-RCF-G1-AG2100 |
| Product Number : G2939A | | Instrument | Run | | 11/16/2015 9:53:27 AM | (GMT +01:00) W. Europe Standard Time | Administrator | V-RCF-G1-AG2100 |
| Name : | | Instrument | Run | | 11/16/2015 9:53:27 AM | (GMT +01:00) W. Europe Standard Time | Administrator | V-RCF-G1-AG2100 |
| Vendor : Agilent Technologies | | Instrument | Run | | 11/16/2015 9:53:27 AM | (GMT +01:00) W. Europe Standard Time | Administrator | V-RCF-G1-AG2100 |
| Serial# : DE72901399 | | Instrument | Run | | 11/16/2015 9:53:27 AM | (GMT +01:00) W. Europe Standard Time | Administrator | V-RCF-G1-AG2100 |
| Firmware : C.01.069 | | Instrument | Run | | 11/16/2015 9:53:27 AM | (GMT +01:00) W. Europe Standard Time | Administrator | V-RCF-G1-AG2100 |
| Cartridge : Electrode | | Instrument | Run | | 11/16/2015 9:53:26 AM | (GMT +01:00) W. Europe Standard Time | Administrator | V-RCF-G1-AG2100 |

 © Copyright 2003 - 2009 Agilent Technologies, Inc.
